# 14-3-3 λ suppresses ethylene-mediated root growth inhibition through EIN3/EIL1 in Arabidopsis

**DOI:** 10.1093/plphys/kiag229

**Published:** 2026-04-21

**Authors:** Zhi-Xin Xiang, Yong-Lun Lv, Ying-Rui Li, Yi-Feng Zhou, Yan-Ke Lu, Meng Xu, Feng Ding

**Affiliations:** Hubei Key Laboratory of Biological Resources Protection and Utilization, Hubei Minzu University, Enshi 44500, China; State Key Laboratory of Hybrid Rice, College of Life Science, Wuhan University, Wuhan 430072, China; Institute of Plant and Food Science, Department of Biology, School of Life Sciences, Southern University of Science and Technology, Shenzhen, Guangdong 518055, China; State Key Laboratory of Hybrid Rice, College of Life Science, Wuhan University, Wuhan 430072, China; Hubei Key Laboratory of Biological Resources Protection and Utilization, Hubei Minzu University, Enshi 44500, China; Hubei Key Laboratory of Biological Resources Protection and Utilization, Hubei Minzu University, Enshi 44500, China; Hubei Key Laboratory of Biological Resources Protection and Utilization, Hubei Minzu University, Enshi 44500, China; State Key Laboratory of Hybrid Rice, College of Life Science, Wuhan University, Wuhan 430072, China; Institute of Plant and Food Science, Department of Biology, School of Life Sciences, Southern University of Science and Technology, Shenzhen, Guangdong 518055, China

## Abstract

Plant roots explore the soil in search of water and nutrients essential for growth. Ethylene-insensitive 3 (EIN3) and EIN3-LIKE 1 (EIL1), the central transcription factors in the ethylene signaling pathway, orchestrate a wide range of developmental and stress-responsive processes, including root growth; however, how transcriptional activation of EIN3/EIL1 is regulated remains to be elucidated. Here, we show that the Arabidopsis (*Arabidopsis thaliana*) regulatory protein 14-3-3 λ, a member of the evolutionarily conserved 14-3-3 protein family, physically interacts with EIN3/EIL1 to attenuate their transcriptional activity, thereby repressing ethylene-mediated inhibition of primary root elongation. Loss of 14-3-3 λ confers hypersensitivity to 1-aminocyclopropane-1-carboxylic acid (ACC), and this phenotype is suppressed by *ein3/eil1* mutations. Notably, ACC treatment promotes the translocation of 14-3-3 λ from the nucleus to the cytosol, which weakens the interaction between 14-3-3 λ and EIN3/EIL1, relieving the repression of EIN3/EIL1 transcriptional activity in plants. Collectively, these findings reveal that 14-3-3 λ dampens the ethylene response by binding to and inhibiting EIN3/EIL1. ACC disrupts this interaction, releasing EIN3/EIL1 to activate ethylene signaling and consequently inhibiting primary root elongation.

## Teaser

Binding of a plant protein to key transcription factors dampens the ethylene signal, preventing roots from halting their growth.

## Introduction

Unlike animals, plants possess neither locomotive agility nor a centralized nervous system, remaining sessile for life. Consequently, they are inescapably subjected to environmental adversities. To survive and reproduce, they have evolved a sophisticated arsenal of stress-resistance mechanisms. As an important component of plants, the root system not only to anchor the shoot and acquire water and nutrients but also to perceive and integrate an array of internal and external cues. By processing these signals, roots orchestrate physiological processes in the most favorable way, allowing the plant to thrive in ever-changing surroundings (Chen et al. 2016; Qin et al. 2020). Ethylene, a volatile plant hormone, both curbs primary root elongation and inhibits gravitropism and lateral root initiation, yet simultaneously stimulates root hair formation (Buer et al. 2006; Růžička et al. 2007; Negi et al. 2008; Qiu et al. 2021; Khoury et al. 2024).

Currently, the biosynthetic pathway of ethylene is well understood; methionine is converted into S-adenosylmethionine (SAM) by the catalysis of S-adenosylmethionine synthetase (SAMS). Subsequently, SAM is catalyzed by ACC synthase (ACSs) to produce 5′-methylthioadenosine (MTA) and 1-aminocyclopropane-1-carboxylic acid (ACC). Finally, the generated ACC is oxidized by ACC oxidase (ACO) to produce ethylene (Hamilton et al. 1991; Tsuchisaka et al. 2009; Park et al. 2021; Pattyn et al. 2021). Once ethylene is synthesized, it is sensed by a family of endoplasmic reticulum-located receptors (ETR). Upon binding ethylene, ETR is inactivated. The inactivated receptor cannot bind CONSTITUTIVE TRIPLE RESPONSE 1 (CTR1), leading to the inability of CTR1 to phosphorylate the endoplasmic reticulum-anchored protein ethylene-insensitive 2 (EIN2). The C-terminal of EIN2 is cleaved and transported into the nucleus, triggering the accumulation of the transcription factor family EIN3 and 5 EILs (EIN3-like proteins), which, in turn, regulate the expression of downstream transcription factors ERFs (Kieber et al. 1993; Alonso et al. 1999; Ju et al. 2012; Qiao et al. 2012; Wen et al. 2012; Binder 2020). Among the 5 EILs, EIL1 exhibits the highest homology to EIN3 and is functionally the most similar (Chao et al. 1997; An et al. 2010). Studies have found that *ein3/eil1*, like *ein2*, can display a completely ethylene-insensitive phenotype during the etiolated seedling stage and in adult plants (Alonso et al. 2003). Conversely, overexpression of *EIN3* or *EIL1* leads to an ethylene-responsive phenotype in *Arabidopsis*, indicating that EIN3 and EIL1 are responsible for the majority of ethylene signal transduction and are core factors in the ethylene signaling pathway (Chao et al. 1997; Guo and Ecker 2003). During the growth and development of roots, ethylene plays a crucial role. Treatment with ethylene gas or the ethylene precursor ACC leads to the inhibition of primary root growth (Harkey et al. 2018). Mutants with reduced sensitivity to ethylene, including *etr1*, *ein2*, *ein3*, and *eil1* in *Arabidopsis*, as well as *Nr* and *gr* in tomato, exhibit increased primary root growth rates (Stepanova et al. 2007; Swarup et al. 2007; Negi et al. 2010). Conversely, in mutants with enhanced ethylene synthesis or signaling, such as *eto1*, *ctr1*, *etr1-6*, *etr1-7*, and *etr2-3,* primary root elongation is reduced (Kieber et al. 1993; Woeste et al. 1999; Harkey et al. 2018). Furthermore, by treating seedlings with ethylene biosynthesis inhibitors AVG and PZA, the inhibitory effect of ethylene on root growth was significantly reduced (Sun et al. 2017). The above studies indicate that ethylene inhibits the growth of plant primary root.

Ethylene response factor *1* (*ERF1*) is a downstream target in the ethylene signaling pathway, directly regulated by EIN3 at the transcriptional level (Solano et al. 1998). It is well known that ERF1 functions in salt, drought, heat stress, and defense response (Lorenzo et al. 2003; Cheng et al. 2013). In addition, ERF1 restrains primary root elongation and dark-grown hypocotyl growth. This has been confirmed by the production of transgenic plants with constitutively activated *ERF1*, which displayed phenotypes similar to that are observed in *ctr1* mutant, *EIN3*-overexpressing plants, and wild-type plants treated with ethylene (Kieber et al. 1993; Chao et al. 1997; Solano et al. 1998). ERF1 can directly upregulate the expression of *ASA1* by binding to the promoter of *ASA1*. ASA1 is a rate-limiting enzyme in auxin biosynthesis, thereby leading to the accumulation of auxin and ethylene-induced root growth inhibition (Mao et al. 2016; Qin et al. 2020).

The 14-3-3 proteins constitute a highly conserved family present across eukaryotes but absent in prokaryotes (Ferl et al. 2002). In Arabidopsis (*Arabidopsis thaliana*), 13 isoforms have been identified, mainly localized in the cytoplasm, where they interact with phosphorylated target proteins to modulate their stability, localization, and interactions, thereby regulating a broad range of biological processes (Aducci et al. 2002; Huang et al. 2022). These processes include flowering, chloroplast development, stomatal movement, seed development, cell growth, and stress responses, with over 300 interactors being characterized (Aducci et al. 2002; Stevers et al. 2017); 14-3-3 proteins also regulate hormone signaling pathways, notably ethylene, as demonstrated by isoforms such as 14-3-3 ψ, which destabilizes ACS to reduce ethylene synthesis, and 14-3-3 ω, which stabilizes ACS to promote ethylene production (Yoon and Kieber 2013; Catala et al. 2014). Beyond their classical phospho-dependent interactions, 14-3-3 proteins also exhibit phosphorylation-independent binding modes that expand their functional repertoire. For example, 14-3-3 binds a non-canonical site within iRhom2's N-terminal domain, where pathogenic mutations disrupt this interaction, leading to deregulated ADAM17 activity and aberrant EGFR signaling implicated in cancer (Bläsius et al. 2024). Similarly, 14-3-3 ζ interacts with Tau protein via electrostatic forces without phosphorylation, enhancing Tau solubility and inhibiting its pathological aggregation relevant to neurodegenerative diseases (Hochmair et al. 2025). Additional phosphorylation-independent cases include 14-3-3 β's interaction with ChREBP stabilized by AMP (Stevers et al. 2017) and pathogen effector XopQ's hijacking of 14-3-3 to suppress plant immunity (Teper et al. 2014). Collectively, these non-canonical interactions highlight 14-3-3 proteins as versatile molecular scaffolds integrating diverse regulatory signals. In plants, 14-3-3 proteins significantly influence root development, a critical aspect of nutrient acquisition and overall growth. Mutants lacking *14-3-3 μ* and *υ* isoforms exhibit inhibited root elongation under red light (Mayfield et al. 2012), while overexpression of bamboo-derived *Pe14-3-3 b* in *Arabidopsis* and of *14-3-3 μ* in tobacco both enhances root growth and H⁺-ATPase activity under stress conditions such as low phosphate availability (He et al. 2015; Guo et al. 2022a). Despite these advances, the molecular mechanisms underlying 14-3-3-mediated regulation of root growth remain elusive.

In this study, we demonstrated that 14-3-3 λ is an EIN3/EIL1-interacting protein that negatively regulates ethylene-repressed root elongation by suppressing the transcriptional activity of EIN3/EIL1 on the *ERF1* promoter. Loss of 14-3-3 λ results in hypersensitivity to ACC, whereas its overexpression leads to reduced sensitivity. 14-3-3 λ physically interacts with EIN3/EIL1 to interfere with their transcriptional activation of *ERF1*; ACC treatment weakens this interaction, thereby activating ethylene signaling and inhibiting root elongation.

## Results

### 14-3-3 λ interacts with EIN3 and EIL1

**Figure 1:**
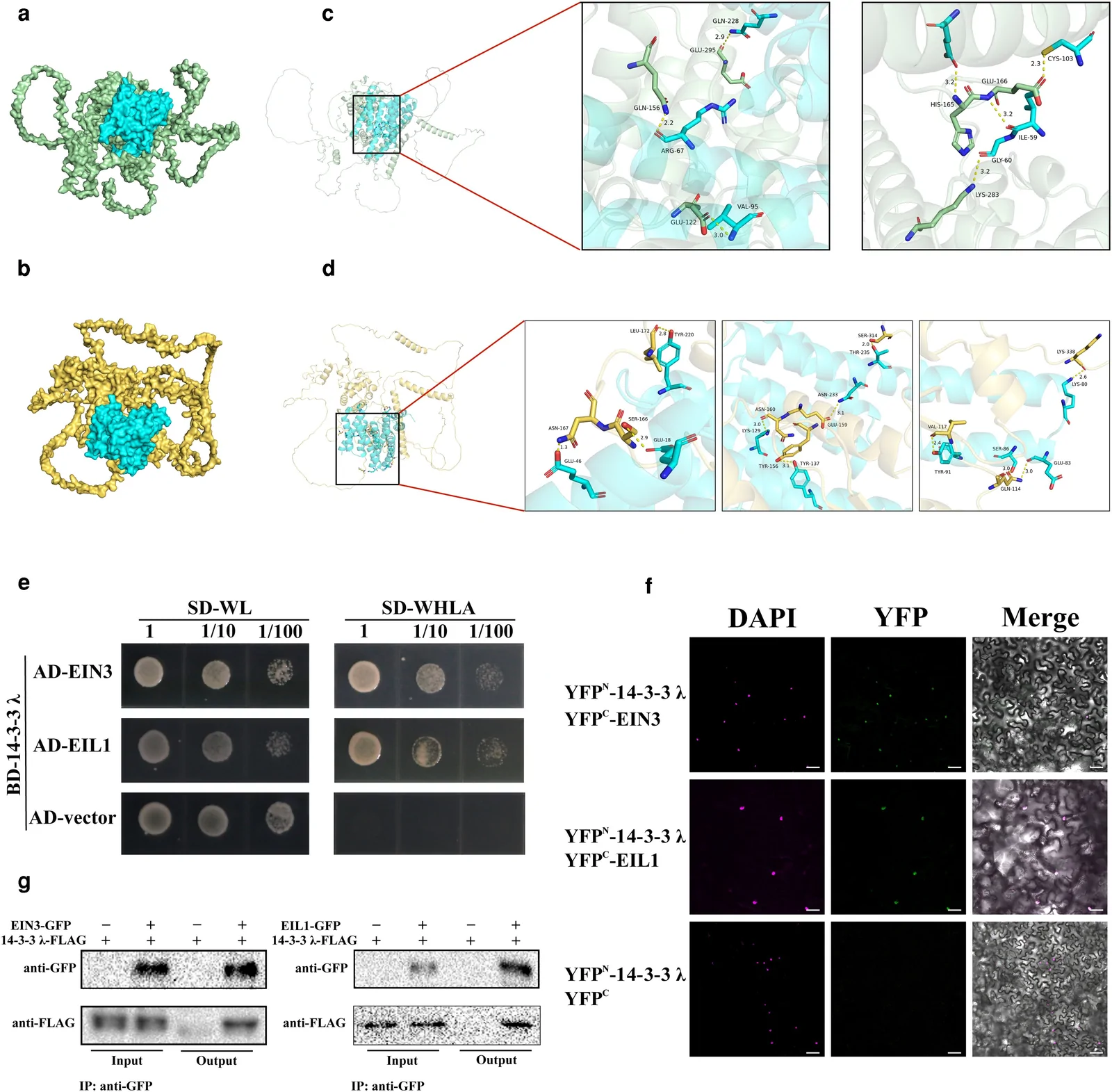
Comparative structural and experimental validation of 14-3-3 λ interactions with EIN3 and EIL1. (a) and (b) Structural models of the complexes formed by 14-3-3 λ with EIN3 (a) and EIL1 (b), as predicted by AlphaFold. In these representations, EIN3 is depicted in blue, EIL1 in yellow, and 14-3-3 λ in cyan. (c) and (d) Close-up views of the major interaction interfaces between 14-3-3 λ and EIN3 (c), as well as 14-3-3 λ and EIL1 (d), highlight the specific molecular interactions predicted by AlphaFold. EIN3 and EIL1 are shown in blue and yellow, respectively. 14-3-3 λ is shown in cyan. Key residues at the binding interfaces are illustrated in stick format (right). (e) Yeast 2-hybrid assays showing the interactions between 14-3-3 λ and EIN3 or EIL1.Transformed yeast cells were grown on SD-WL (without tryptophan and leucine) and selective medium SD-WLHA (without tryptophan, leucine, histidine, and adenine). AD, activation domain; BD, DNA-binding domain. **(**f**)** BiFC analysis. 14-3-3 λ was fused with the N-terminal fragment of YFP to form YFP^N^-14-3-3 λ. EIN3 and EIL1 were fused with the C-terminal fragment of YFP to form YFP^C^-EIN3 and YFP^C^-EIL1, respectively. YFP fluorescence was detected in *N. benthamiana* leaves co-infiltrated with the combination of indicated constructs. The positions of nuclei were shown by 4′,6-diamidino-2-phenylindole (DAPI) staining. **(**g**)** Co-IP analysis. Anti-GFP antibody was used to immunoprecipitated EIN3/EIL1 and their interacting proteins, and the immunoprecipitated proteins were analyzed by western blotting with anti-FLAG antibody and anti-GFP antibody, respectively.

The physiological response to ethylene is typically characterized by rapid growth inhibition, such as the suppression of primary root elongation, a process largely governed by the master transcriptional regulator EIN3 (Binder et al. 2004; Růžička et al. 2007; Mao et al. 2016). We hypothesized that the identification of EIN3-interacting proteins will advance our understanding of the sophisticated regulatory mechanisms underlying EIN3 function in primary root growth and ethylene signaling. To identify EIN3 partners in *Arabidopsis*, yeast 2-hybrid (Y2H) screening assay was performed. After screening the normalized cDNA library, a fragment of 14-3-3 λ was identified as a candidate interactor. Structural predictions using artificial intelligence (AI), such as AlphaFold, offer powerful tools for discovering protein-protein interactions (Abramson et al. 2024; Homma et al. 2024). We then performed protein-protein docking simulations using AlphaFold to predict the interaction between 14-3-3 λ and both EIN3 and its homolog EIL1. The results indicated that 14-3-3 λ interacts with both EIN3 and EIL1 (Fig. 1a and b). Furthevrmore, visualization of the EIN3-14-3-3 λ complex using PyMOL revealed detailed interaction patterns, highlighting the amino acid residues involved in the binding interface. These residues formed multiple strong hydrogen bonds, salt bridges, and hydrophobic interactions. Notably, the hydrogen bond lengths were significantly shorter than the typical 3.5 Å, indicating a highly stable and specific interaction (Fig. 1c and d). To validate the predicted interactions, we cloned full-length coding sequence (CDS) of 14-3-3 λ and re-examined its interaction between EIN3/EIL1 in yeast cells, our results showed that 14-3-3 λ and EIN3/EIL1 indeed had interaction in yeast cells (Fig. 1e). Furthermore, we performed biomolecular fluorescence complementation (BiFC) assays by constructing YFP^N^-14-3-3 λ, YFP^C^-EIN3/EIL1. In *N. benthamiana* leaf epidermal cells, co-expression of YFP^N^-14-3-3 λ with YFP^C^-EIN3 or YFP^C^-EIL1 reconstructed YFP fluorescence signals in the nucleus, whereas no fluorescence was observed in the negative control (Fig. 1f), providing strong evidence for direct interactions between 14-3-3 λ and EIN3/EIL1 in planta. Additionally, co-immunoprecipitation (Co-IP) assays were conducted by co-expressing *35S::14-3-3 λ-FLAG* and *35S::EIN3-GFP* or *35S::EIL1-GFP* in *N.benthamiana*. Total protein extracts were immunoprecipitated using an anti-GFP antibody, and the presence of 14-3-3 λ-FLAG was detected in the immunoprecipitates, confirming the physical interactions between 14-3-3 λ and EIN3/EIL1 (Fig. 1g). All the above results collectively demonstrate the interaction between 14-3-3 λ and EIN3/EIL1.

### Domain and motif analysis of 14-3-3 proteins

**Figure 2:**
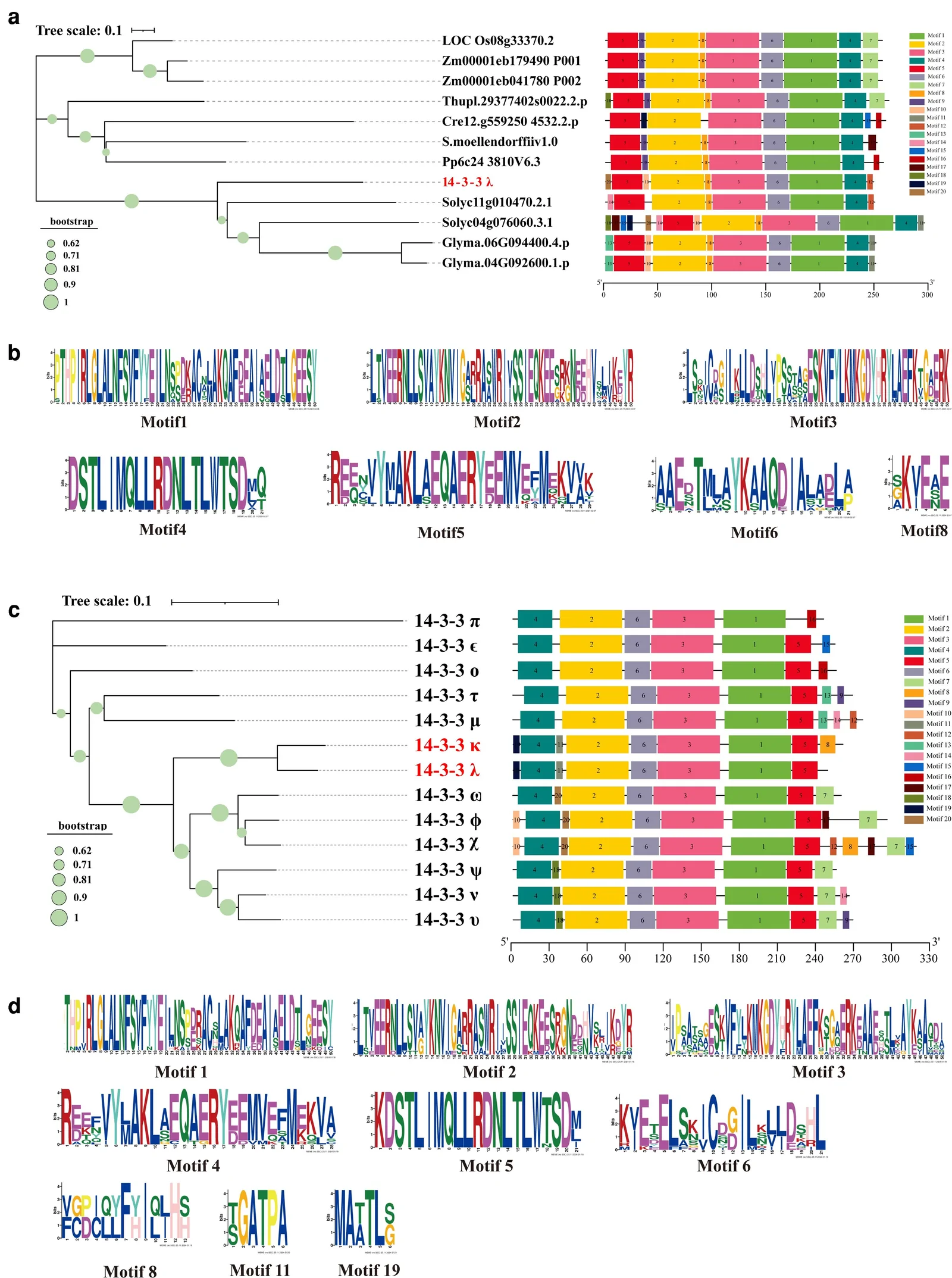
Phylogenetic relationships and motif analysis of 14-3-3 proteins. (a) The phylogenetic relationships of 14-3-3 λ in *Chlamydomonas reinhardtii*, *Physcomitrium patens, Selaginella moellendorffii*, *Thuja plicata*, *Arabidopsis thaliana*, *Solanum lycopersicum*, *Oryza sativa*, *Glycine max*, and *Zea mays*. The motifs listed on the right are sorted according to their frequency of occurrence in these 14-3-3 λ proteins. (b) Motifs that fall into 14-3-3 λ domains are motif 1, 2, 3, 4, 5, 6, and 8. Height or occupancy of letters represents the conservation ratio of certain amino acid residues. (c) and (d) Phylogenetic relationships and motif analysis of 13 *Arabidopsis* 14-3-3 proteins. The scale bar represents 0.1 amino acid substitutions per site. Numbers at the nodes in (a) and (c) indicate bootstrap support values (based on 1,000 replicates). Values range from 0 to 1, with higher values indicating greater statistical support for the branch.

The similarity in protein sequences and domains reflects the conservation of their functions. To better understand the functions of 14-3-3 λ protein, we first utilized bioinformatics to analyze the evolutionary conservation of the 14-3-3 λ protein across different species, including *Chlamydomonas reinhardtii*, *Physcomitrium patens, Selaginella moellendorffii, Thuja plicata*, *Arabidopsis thaliana*, *Solanum lycopersicum*, *Oryza sativa*, *Glycine max*, and *Zea mays*. We found that the 14-3-3 λ protein is highly conserved during evolution, with motifs 1, 2, 3, 4, 5, 6, and 8 being the shared domains among them (Fig. 2a and b). Subsequently, we also analyzed the amino acid sequences and functional domains of the 13 members of the 14-3-3 λ family in *Arabidopsis thaliana*. We discovered that 14-3-3 λ exhibits the highest homology with 14-3-3 κ, with the difference being that the C-terminus of the 14-3-3 κ protein contains an additional Motif 8 compared to 14-3-3 λ (Fig. 2c and d). A detailed study of specific motifs and domains within the 14-3-3 members will help reveal the potential working mechanisms of the 14-3-3 λ in controlling plant growth and stress responses. These results indicate that the 14-3-3 λ protein is evolutionarily conserved and may play a role in numerous biological processes.

### Plants lacking 14-3-3 λ function are more sensitivity to ACC treatment

Previous studies have demonstrated that ethylene or its precursor ACC reduce root elongation in a concentration-dependent manner by inhibition of the cell elongation process (Li et al. 2015; Mao et al. 2016). The interaction of 14-3-3 λ with EIN3/EIL1 prompted us to test whether it functions in ethylene-mediated inhibition of primary root elongation. To achieve this, we utilized previously reported T-DNA insertion mutant of *14-3-3 λ*, *14-3-3 κ*, and *14-3-3 κ λ* double mutants (Zhang et al. 2019a) and assayed their primary root growth under ACC treatment. In seed plants, the conversion of the ethylene precursor ACC to ethylene is catalyzed by ACO, thereby making ACC treatment a commonly employed method for eliciting ethylene response (Yoon and Kieber 2013). Under the control condition, the primary root length of both the wild type (WT), *14-3-3 λ*, and *14-3-3 κ* was comparable. However, upon treatment with varying concentrations of ACC (0.1 µM, 1 µM, and 10 µM), *14-3-3 κ* consistently exhibited similar primary root length phenotype to WT, while *14-3-3 λ* displayed significantly shorter primary root lengths relative to WT (Figure S1a and b). Additionally, we observed that ACC treatment had no effect on the mRNA levels of *14-3-3 λ* or *14-3-3 κ* in the WT (Figure S1c). To further investigate whether ACC treatment affects the protein accumulation of 14-3-3 λ, we generated 14-3-3 λ-specific antibodies by immunizing a rabbit with *Escherichia coli*-expressed 14-3-3 λ. The specificity of the antibodies was validated using immunoblot analysis with protein extracts from WT, *14-3-3 λ*, *14-3-3 κ*, and *14-3-3 λ κ* double mutant. Total proteins were extracted from 7-day-old WT, single, and double mutant seedlings and analyzed by immunoblots with 14-3-3 λ antibodies. Strong cross-reactivity was observed in WT and *14-3-3 κ*, weak cross-reactivity in *14-3-3 λ*, and no detectable signal in the *14-3-3 λ κ* double mutant (Figure S2). Utilizing anti-14-3-3 λ detection, we found that ACC treatment did not alter the accumulation of 14-3-3 λ protein (Figure S1d), indicating that ethylene does not regulate 14-3-3 λ at the post-transcriptional level. The above results indicated that 14-3-3 λ rather than 14-3-3 κ is involved in ethylene-mediated inhibition of primary root elongation.

To explain this phenotypic difference, we also utilized AlphaFold to analyze the interaction between 14-3-3 κ and EIN3. Structural modeling analysis revealed that in the interaction interface between 14-3-3 κ and EIN3, the majority of the formed hydrogen bonds were longer than 3.5 Å, which are typically not considered effective hydrogen bonds (Figure S3a and b). In contrast, the interaction interface between 14-3-3 λ and EIN3 formed short-range strong hydrogen bonds of 2.2 to 3.0 Å (Fig. 1c). This significant difference in hydrogen bond distance may be a factor contributing to the much weaker interaction strength between 14-3-3 κ and EIN3 compared to that between 14-3-3 λ and EIN3. The statistical results of binding energy demonstrate that 14-3-3 λ/EIN3 exhibits a much higher binding energy (∼28 kcal/mol) than 14-3-3 κ/EIN3 (∼13 kcal/mol), further corroborating the aforementioned speculation (Figure S3c). In addition, the results of Y2H, Co-IP, and BiFC assays showed that 14-3-3 κ does not interact with EIN3 (Figure S3d—f). Therefore, the inability of 14-3-3 κ to interact with EIN3/EIL1, despite being the closest homolog of 14-3-3 λ, likely underpins this functional divergence. Notably, the *14-3-3 λ κ* double mutant exhibited a phenotype resembling that of *14-3-3 λ* under ACC treatment, further reinforcing this conclusion (Figure S1a and b).

**Figure 3:**
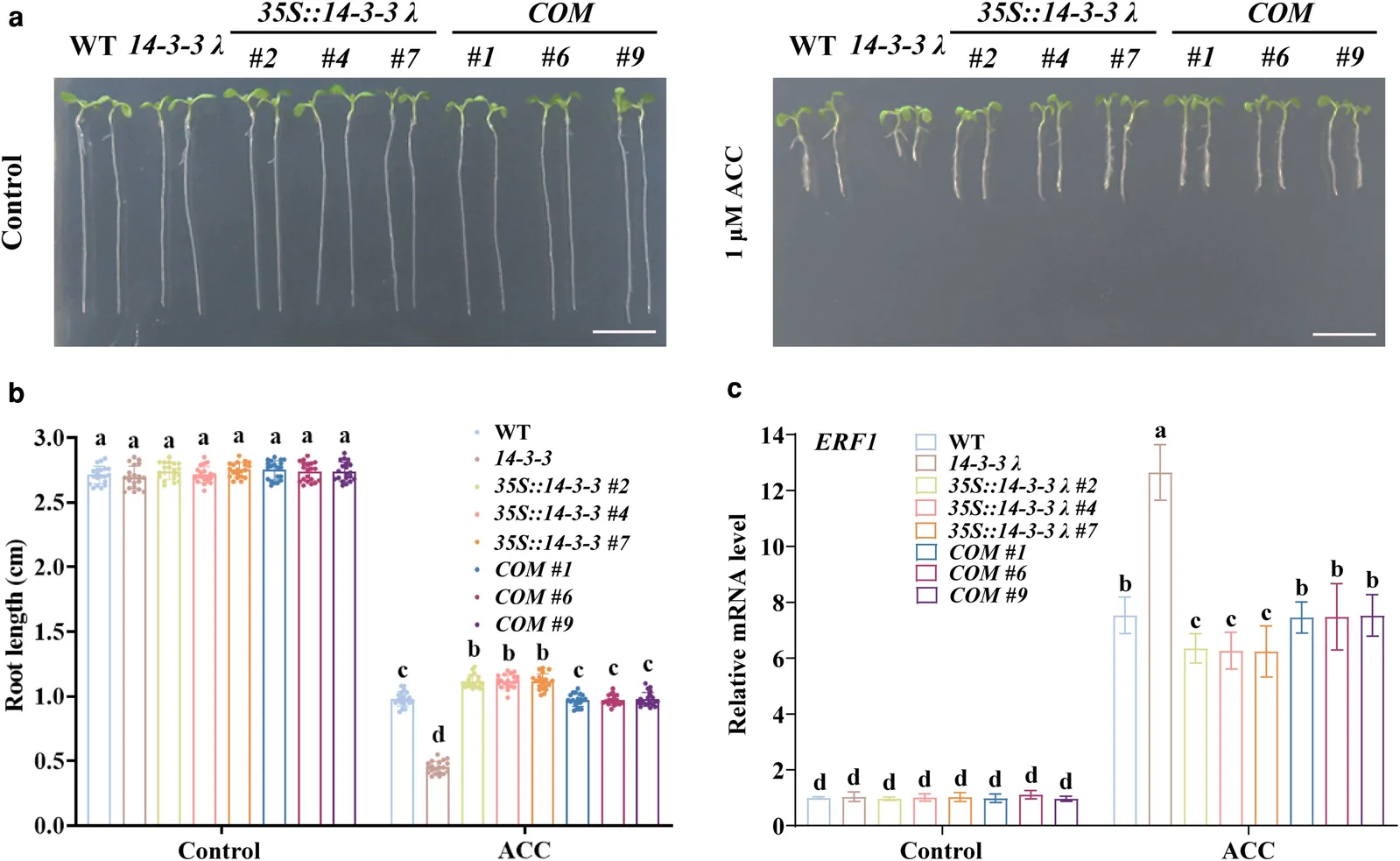
Plants lacking 14-3-3 λ function are more sensitive to ACC treatment. (a) The primary root elongation phenotype of wild-type (WT), *14-3-3 λ*, *35S::14-3-3 λ* (*#2*, *#4*, *#7*) lines and complementary lines (*COM #1*, *#6*, *#9*). Seedlings were grown on 1/2 MS medium supplemented with or without 1 μM ACC for 7 days. Scale bar = 1 cm. (b) Quantitative analysis of primary root length in WT, *14-3-3 λ*, *35S::14-3-3 λ*, and *COM*. Root lengths were measured 7 days after germination on 1/2 MS medium supplemented with or without 1 μM ACC. (c) Relative expression levels of *ERF1* were analyzed by RT-qPCR in WT, *14-3-3 λ*, *35S::14-3-3 λ*, and *COM*. Data are presented as means ± SD from 3 independent biological replicates. Data represent mean ± SD (n = 20). Different letters indicate statistically significant differences (*P* < 0.05) as determined by 1-way ANOVA followed by Tukey's multiple comparison test.

Subsequently, we tried to complement *14-3-3 λ* mutant with the full-length coding sequence of *14-3-3 λ* driven under its native promoter to confirm that the mutant phenotypes were caused by the loss of *14-3-3 λ*. As expected, these complemental lines (*COM #1, #6, #9*) had similar primary root length as the WT under ACC treatment (Fig. 3a and b). We also tested the ethylene sensitivity of the *14-3-3 λ*-overexpressing transgenic lines (*35S::14-3-3 λ #2, #4, #7*), where the expression of *14-3-3 λ* was driven by the 35S promoter (Zhang et al. 2019a). In contrast to the *14-3-3 λ* mutant, *35S::14-3-3 λ* displayed an enhanced insensitivity to ethylene, as evidenced by slightly longer primary roots compared to the WT in the presence of ACC (Fig. 3a and b), further validating the negative regulation of *14-3-3 λ* in ethylene-inhibited primary root growth. *ERF1* is a downstream target in the ethylene signaling pathway, directly regulated by EIN3 at the transcriptional level. Previous studies have demonstrated that EIN3 binds to the *ERF1* promoter to activate its expression, thereby contributing to ethylene-induced inhibition of root growth (Solano et al. 1998; Mao et al. 2016). ACC treatment induced *ERF1* expression in WT, and this induction was significantly enhanced in the *14-3-3 λ* mutant, while it was suppressed in *35S::14-3-3 λ* lines (Fig. 3c), consistent with the observed changes in primary root growth. All the above results indicate that 14-3-3 λ represses ethylene-mediated inhibition of primary root elongation.

### 14-3-3 λ does not affect ethylene biosynthesis and EIN3 protein abundance

**Figure 4:**
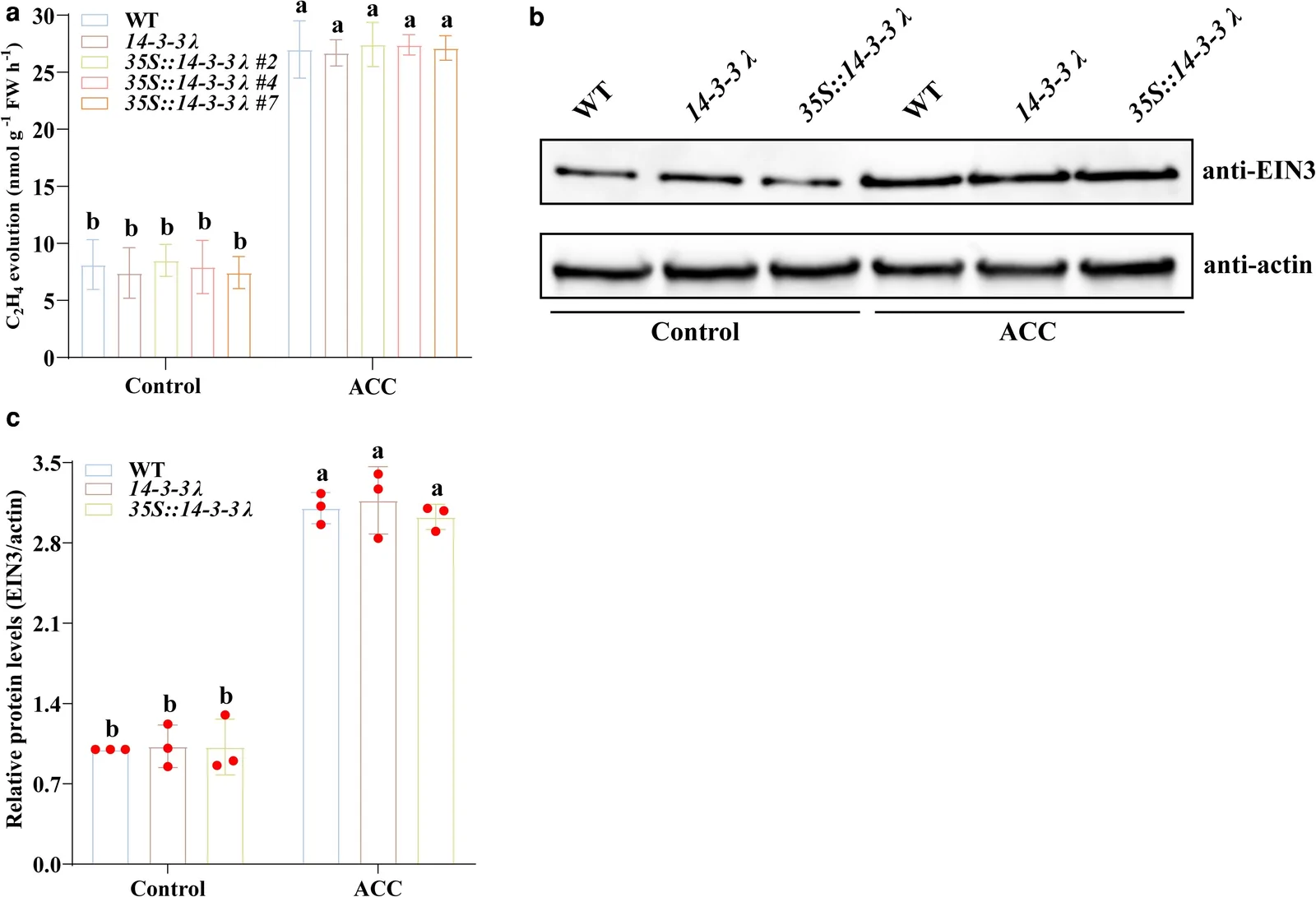
14-3-3 λ does not affect ethylene biosynthesis and EIN3 protein accumulation. (a) Ethylene evolution from roots of 5-d-old WT, *14-3-3 λ*, and *35S::14-3-3 λ* plants upon exposure to 10 μM ACC for 12 h. (b) Five-day-old light-grown seedlings (WT, *14-3-3 λ*, and *35S::14-3-3 λ*) were treated with 10 μM ACC for 12 h. Total protein extracts were probed with anti-EIN3 or anti-actin antibody. (c) The relative EIN3 protein levels were quantified by measuring the intensity of each immunoblot band using ImageJ. These levels were normalized to the actin loading control and then further calibrated against the untreated WT plants, which were set to 1. Data are presented as means ± SD from 3 independent biological replicates. Different letters indicate statistically significant differences (*P* < 0.05) as determined by 1-way ANOVA followed by Tukey's multiple comparison test.

14-3-3 proteins, including 14-3-3 ω, have been implicated in playing a role in ethylene biosynthesis by interacting with ACS in rice, barley, and *Arabidopsis* (Solano et al. 1998; Huang et al. 2013; Yoon and Kieber 2013). To explore whether 14-3-3 λ suppresses ethylene-inhibited primary root growth by affecting ethylene biosynthesis, we examined the expression of core genes *1-aminocyclopropane-1-carboxylic acid synthase 2* (*ACS2*), *ACS5* (*1-aminocyclopropane-1-carboxylic acid synthase 5*), *ACS6* (*1-aminocyclopropane-1-carboxylic acid synthase 6*), *ACS8* (*1-aminocyclopropane-1-carboxylic acid synthase 8*), *ACO1* (*1-aminocyclopropane-1-carboxylic acid oxidase 1*), *ACO2* (*1-aminocyclopropane-1-carboxylic acid oxidase 2*), and *ACO4* (*1-aminocyclopropane-1-carboxylic acid oxidase 4*) (Larsen 2015) in the ethylene biosynthesis pathway in WT, *14-3-3 λ*, and *35S::14-3-3 λ* following ACC treatment. Consistent with previous reports, we observed that ACC treatment significantly induced the expression of these genes. However, regardless of ACC treatment, no obvious differences in the mRNA levels of these ethylene biosynthesis genes were observed among WT, *14-3-3 λ*, and 3*5S::14-3-3 λ* (Figure S4a-g). Furthermore, we directly measured ethylene (C_2_H_4_) evolution in WT, *14-3-3 λ*, and *35S::14-3-3 λ* after ACC treatment. Similarly, our results showed no significant differences in ethylene production (Fig. 4a), suggesting that 14-3-3 λ does not modulate ethylene signaling through affecting ethylene biosynthesis. To explore whether 14-3-3 λ affects EIN3 protein accumulation, we examined EIN3 abundance in the roots of WT, *14-3-3 λ*, and *35S::14-3-3 λ* seedlings and found that EIN3 was unchanged in above seedlings (Fig. 4b and c). Based on these results, we conclude that 14-3-3 λ neither affects ethylene synthesis nor influences the abundance of EIN3 protein.

### 14-3-3 λ interferes with transcriptional activity of EIN3 and EIL1

**Figure 5:**
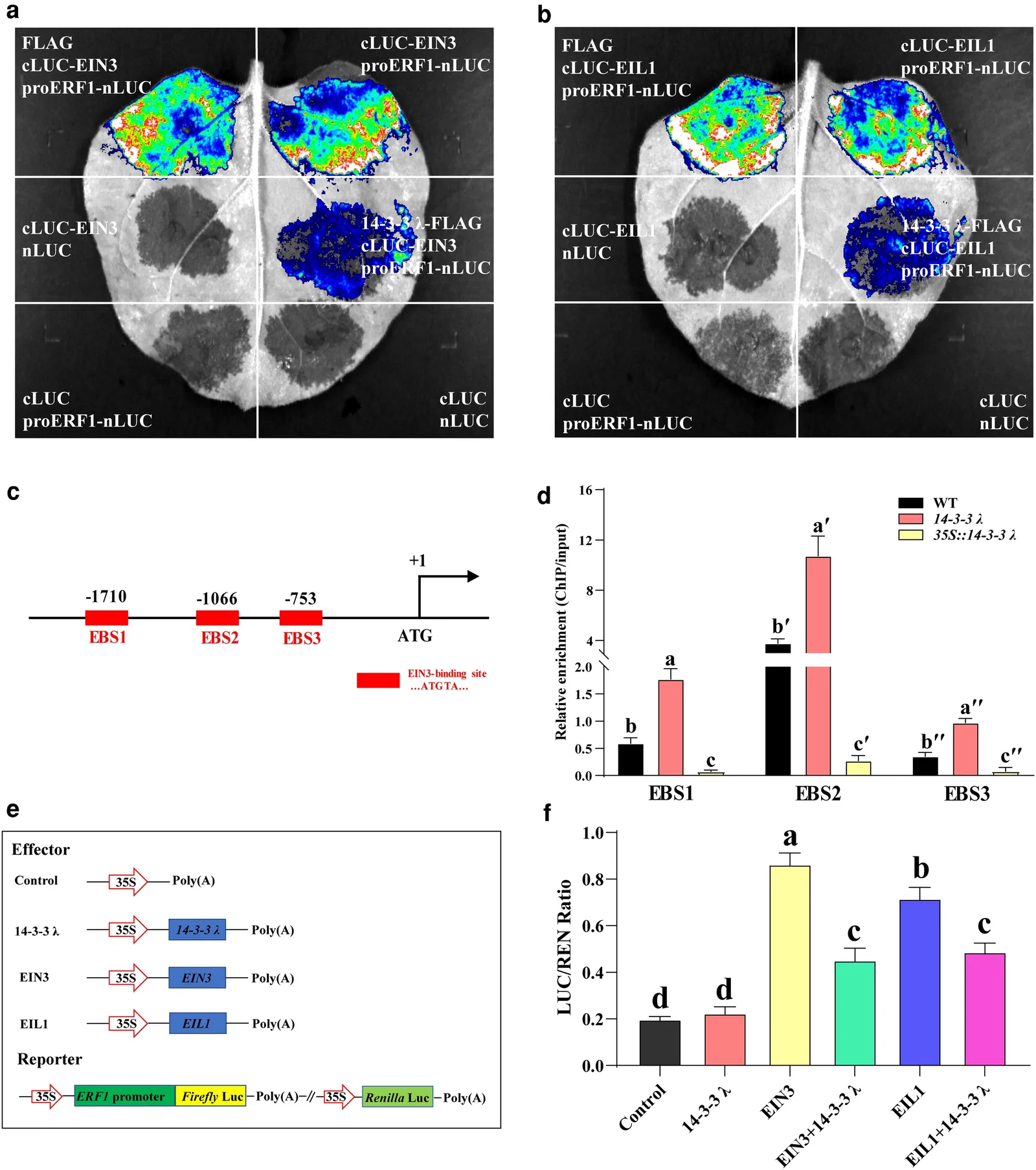
14-3-3 λ impairs EIN3 binding to the *ERF1* promoter and represses EIN3/EIL1-mediated transcriptional activation. (a) and (b**)** Luciferase complementation imaging assays showing the transcriptional activity of EIN3 (a) and EIL1 (b). In (a), co-expression of cLUC-EIN3 with proERF1-nLUC reconstitutes luciferase activity (top left), while co-expression of 14-3-3 λ-FLAG, cLUC-EIN3, and proERF1-nLUC abolishes this activity (middle right). In (b), co-expression of cLUC-EIL1 with proERF1-nLUC reconstitutes luciferase activity (top left), while co-expression of 14-3-3 λ-FLAG, cLUC-EIL1, and proERF1-nLUC abolishes this activity. Negative controls include cLUC-EIN3/EIL1 with nLUC (middle left), cLUC/proERF1-nLUC (bottom left), and cLUC-nLUC (bottom right). (c) and (d) ChIP-qPCR to assay the association of 14-3-3 λ with the EIN3-binding sites on the *ERF1* promoter. Diagrams (c) showing the location of EIN3-binding sites on the *ERF1* promoters. Cross-linked chromatin extracted from 7-day-old WT, *14-3-3 λ*, and *35S::14-3-3 λ* seedlings was immunoprecipitated with anti-EIN3 antibody. The eluted DNA was amplified by quantitative PCR for sequences adjacent to EBS1, EBS2, and EBS3. (e) and (f**)** Dual-luciferase reporter assays were employed to examine the role of 14-3-3 λ in EIN3/EIL1-activated *ERF1* expression. The schematic diagram (e) illustrates the reporter and effector genes used in the assay. The effector and reporter genes were co-expressed in *N. benthamiana* leaves, and the activities of LUC and REN were measured. The relative LUC intensity **(**f**)** represents the ERF1::LUC activity normalized to the internal control (REN driven by the *35S* promoter). Data are presented as means ± SD from 3 independent biological replicates. Different letters indicate statistically significant differences (*P* < 0.05) as determined by 1-way ANOVA followed by Tukey's multiple comparison test.

Because EIN3/EIL1 associates with the promoter of *ERF1* to induce its transcription, we next examined whether 14-3-3 λ could repress the effects of EIN3 and EIL1 on the *ERF1*. We conducted split-LUC assay to validate this hypothesis. Constructs harboring the *ERF1* promoter fused with the N-terminal fragment of LUC (proERF1-nLUC) and the C-terminal fragment of LUC fused with EIN3 (cLUC- EIN3) were co-infiltrated into *N.benthamiana* leaves to transiently co-express these fusion proteins. Our results showed that co-expression of proERF1-nLUC and cLUC-EIN3, or FLAG along with proERF1-nLUC and cLUC-EIN3, resulted in a robust luminescence signal, while the inclusion of 14-3-3 λ-FLAG in the co-expression of proERF1-nLUC/cLUC-EIN3 significantly diminished this luminescence signal (Fig. 5a). Similar results were also observed with EIL1 system (Fig. 5b), indicating that 14-3-3 λ interferes with the interaction between EIN3/EIL1 and the *ERF1* promoter. Previous studies have shown that EIN3 binds to the ethylene response element, which contains the EBS within the *ERF1* promoter (Fig. 5c) (Solano et al. 1998). We then performed chromatin immunoprecipitation followed by quantitative PCR (ChIP-qPCR) to examine the effect of 14-3-3 λ on EIN3 binding to the *ERF1* promoter, and found that the loss of 14-3-3 λ enhances the binding of EIN3 to the *ERF1* promoter, whereas *14-3-3 λ* overexpression inhibits this binding (Fig. 5d). In addition, we performed dual-LUC reporter assays in *N.benthamiana* leaves. In this system, LUC was used as a reporter under the control of the *ERF1* promoter, while Renilla luciferase (REN) driven by the constitutive *35S* promoter served as an internal control (Fig. 5e). Our results demonstrated a substantial increase in LUC activity upon expression of *35S::EIN3* or *35S::EIL1* as effectors. However, LUC activity remained unchanged when *35S::14-3-3 λ* was expressed alone. Importantly, the EIN3/EIL1-induced LUC activity was significantly reduced when *14-3-3 λ* was co-expressed with EIN3 or EIL1 (Fig. 5f). These findings highlight the inhibitory role of 14-3-3 λ in modulating the transcriptional activity of EIN3/EIL1.

### Ethylene dampens the interaction between 14-3-3 λ and EIN3 and releases EIN3 transcriptional activity

**Figure 6:**
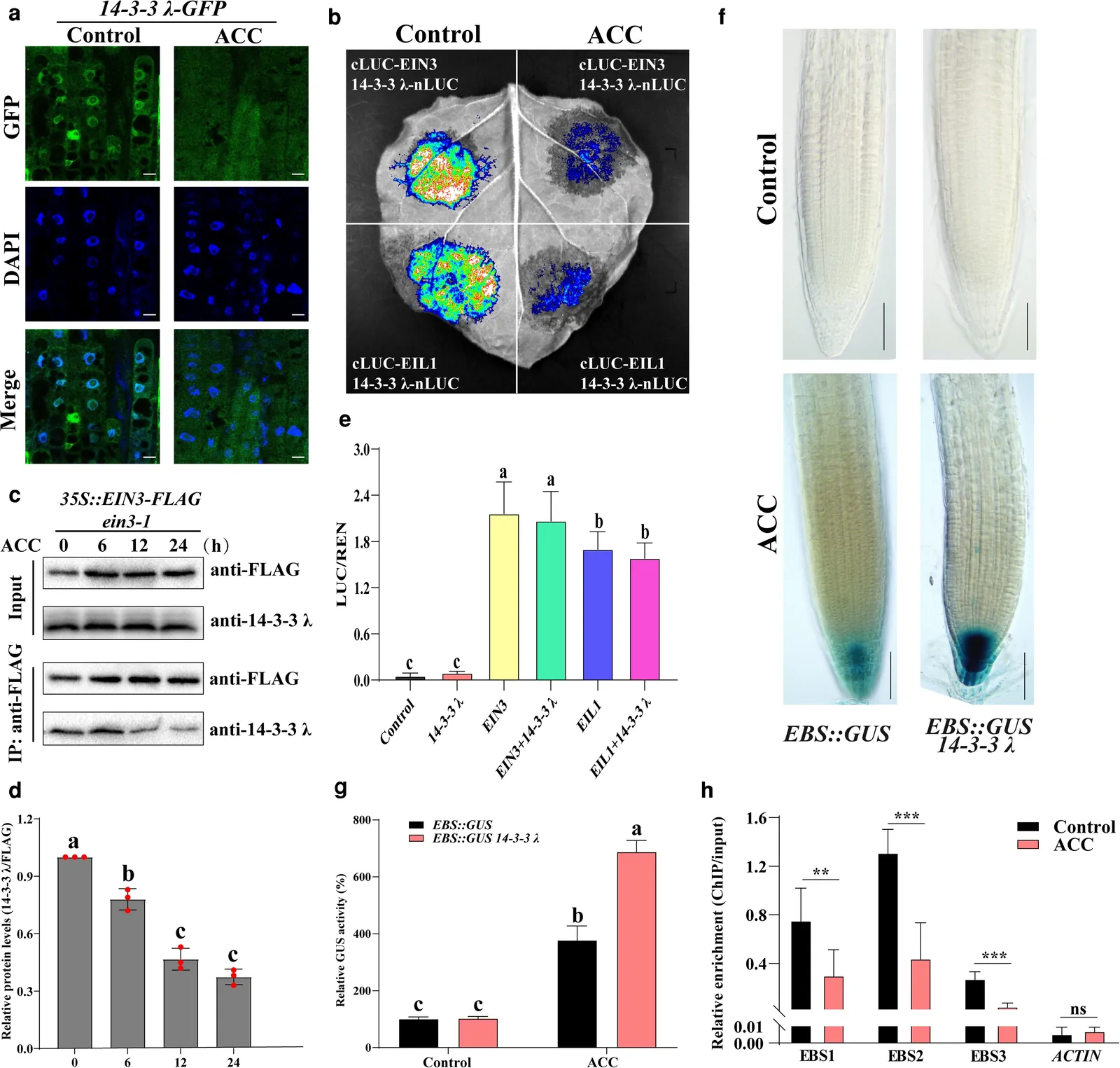
Ethylene dampens the interaction between 14-3-3 λ and EIN3/EIL1 and releases EIN3 transcriptional activity. (a) Subcellular localization of 14-3-3 λ in root cells of *35S:: 14-3-3 λ-GFP* seedlings treated or untreated with ACC for 12 h. Scale bars, 5 μm. (b) The interaction between 14-3-3 λ and EIN3/EIL1 in the presence or absence of ACC was detected using the split LUC assay. The constructs of 14-3-3 λ-nLUC and cLUC-EIN3/EIL1 were transiently expressed in *N. benthamiana* leaves for 2 days, followed by injection of ACC solution into the leaves. LUC images were captured 1 d after injection. (c) Co-IP assays examine the effect of ethylene on the interaction of 14-3-3 λ with EIN3. 10 μM ACC-treated 7-d-old *35S::EIN3-FLAG ein3-1* for 0, 6, 12, 24 h, and then immunoprecipitated total protein with anti-FLAG antibody-coupled agarose. The precipitates were separated by SDS-PAGE and detected with anti-14-3-3 λ antibody. (d) The relative 14-3-3 λ protein levels in the immunoprecipitated fractions were quantified by measuring the intensity of each immunoblot band using ImageJ. These levels were normalized to the anti-FLAG signal and then further calibrated against the ACC-untreated plants, which were set to 1. (e) Dual-LUC reporter assays by incorporating ACC treatment. (f) GUS staining images of *EBS::GUS* and *EBS::GUS 14-3-3 λ* without or with ACC treatment. (g) Relative GUS activity of *EBS::GUS* and *EBS::GUS 14-3-3 λ* without or with ACC treatment. The activity of *EBS::GUS* without ACC treatment was set as 100. (h) ChIP-qPCR analysis showing that ACC treatment increases EIN3 binding to *ERF1* promoter regions (EBS1, EBS2, and EBS3). Cross-linked chromatin extracted from 7-day-old *35S::14-3-3 λ* seedlings treated with or without 10 μM ACC for 12 h was immunoprecipitated with anti-*14-3-3 λ* antibody. The eluted DNA was amplified by quantitative PCR for sequences adjacent to EBS1, EBS2, and EBS3. ACTIN was used as the internal reference gene. Data are presented as means ± SD from 3 independent biological replicates. Different letters indicate statistically significant differences (*P* < 0.05) as determined by 1-way ANOVA followed by Tukey's multiple comparison test. Asterisks indicate significant differences between the indicated columns (Student's *t*-test): **, *P* < 0.01; ***, *P* < 0.001. ns: no significance.

We have demonstrated that 14-3-3 λ negatively regulates the activity of EIN3 through physical interaction; however, it remains unclear whether and how ethylene affects their interaction. 14-3-3 λ is localized in both the cytoplasm and the nucleus (Liu et al. 2017). Considering the interaction between 14-3-3 λ and EIN3 in the nucleus, we next explored whether ACC treatment reduces the inhibitory effect of 14-3-3 λ on EIN3 transcriptional activity by altering the localization of 14-3-3 λ. We first examined whether ACC treatment changes the subcellular localization of 14-3-3 λ. To this end, we generated transgenic plants expressing *35S::14-3-3 λ-GFP* in the wild type. We observed that 14-3-3 λ-GFP signals in both the cytoplasm and nucleus of *35S::14-3-3 λ-GFP* root cells in the absence of ACC (Fig. 6a). However, upon treatment with ACC, 14-3-3 λ-GFP signals were almost undetectable in the nucleus, indicating that ACC induces the nuclear export of 14-3-3 λ protein (Fig. 6a). We also conducted the split-LUC assay in *N. benthamiana* leaves. Constructs encoding 14-3-3 λ-nLUC and cLUC-EIN3 or cLUC-EIL1 were co-infiltrated, followed by monitoring LUC activity after ACC treatment. The results revealed a significant reduction in LUC signal intensity upon ACC treatment compared to the untreated control (Fig. 6b). Furthermore, we obtained similar results through BiFC experiments (Figure S5a and b), indicating that ACC disrupts the interaction between 14-3-3 λ and EIN3/EIL1. To further validate this finding, we conducted Co-IP assays using *35S::EIN3-FLAG* transgenic plants (*ein3-1* background). Plants were either untreated or treated with ACC for 6, 12, or 24 hours, and then, EIN3-FLAG was immunoprecipitated with anti-FLAG antibody–conjugated agarose, and co-immunoprecipitated proteins were probed with anti-14-3-3 λ antibodies. The results revealed a gradual decrease in the amount of co-precipitated 14-3-3 λ protein with sustained ACC treatment (Fig. 6c and d), providing further evidence that ACC disrupts the interaction between 14-3-3 λ and EIN3. If this disruption alleviates the inhibitory effect of 14-3-3 λ on EIN3/EIL1 transcriptional activity, it should be reflected in downstream transcriptional responses. To test this hypothesis, we extended our previously designed dual-LUC reporter assays by incorporating ACC treatmen and found that ACC treatment significantly mitigated the reduction in EIN3/EIL1-induced LUC activity caused by 14-3-3 λ, compared to the untreated control (Fig. 5f, Fig. 6e).

To further confirm this observation, we utilized the *EIN3-Binding Site (EBS)::GUS* reporter, a transcriptional reporter driven by the EIN3-binding cis-element, which is widely used to monitor ethylene signaling activation (Stepanova et al. 2007; Li et al. 2015). We crossed the *14-3-3 λ* mutant with the *EBS::GUS* line and analyzed GUS enzymatic activity under both ACC-treated and untreated conditions. Our results showed that ACC treatment significantly enhanced GUS activity in the *EBS::GUS 14-3-3 λ* line compared to the *EBS::GUS* control (Fig. 6f and g). These findings strongly suggest that ethylene can release EIN3 transcriptional activity from suppression by 14-3-3 λ. If this holds true in plants, the association of 14-3-3 λ with EIN3-targeted genes should also be disrupted by ACC treatment. To investigate this hypothesis, we performed ChIP-qPCR to examine the enrichment of 14-3-3 λ on EBS regions of the *ERF1* promoter. In the absence of ACC, 14-3-3 λ exhibited a strong association with the *ERF1* promoter DNA. However, ACC treatment significantly reduced this association (Fig. 6h). These findings provide evidence that ACC reduces the interaction between 14-3-3 λ and EIN3 by inducing the nuclear export of 14-3-3 λ, thereby alleviating its inhibitory effects on ethylene signaling. Together, these results uncover a critical regulatory mechanism in which 14-3-3 λ binding restrains EIN3/EIL1 activity under inactive ethylene signaling but allows its activation upon ethylene signaling activation.

### 14-3-3 λ act upstream of EIN3/EIL1 to regulate ethylene-inhibited primary root growth

**Figure 7:**
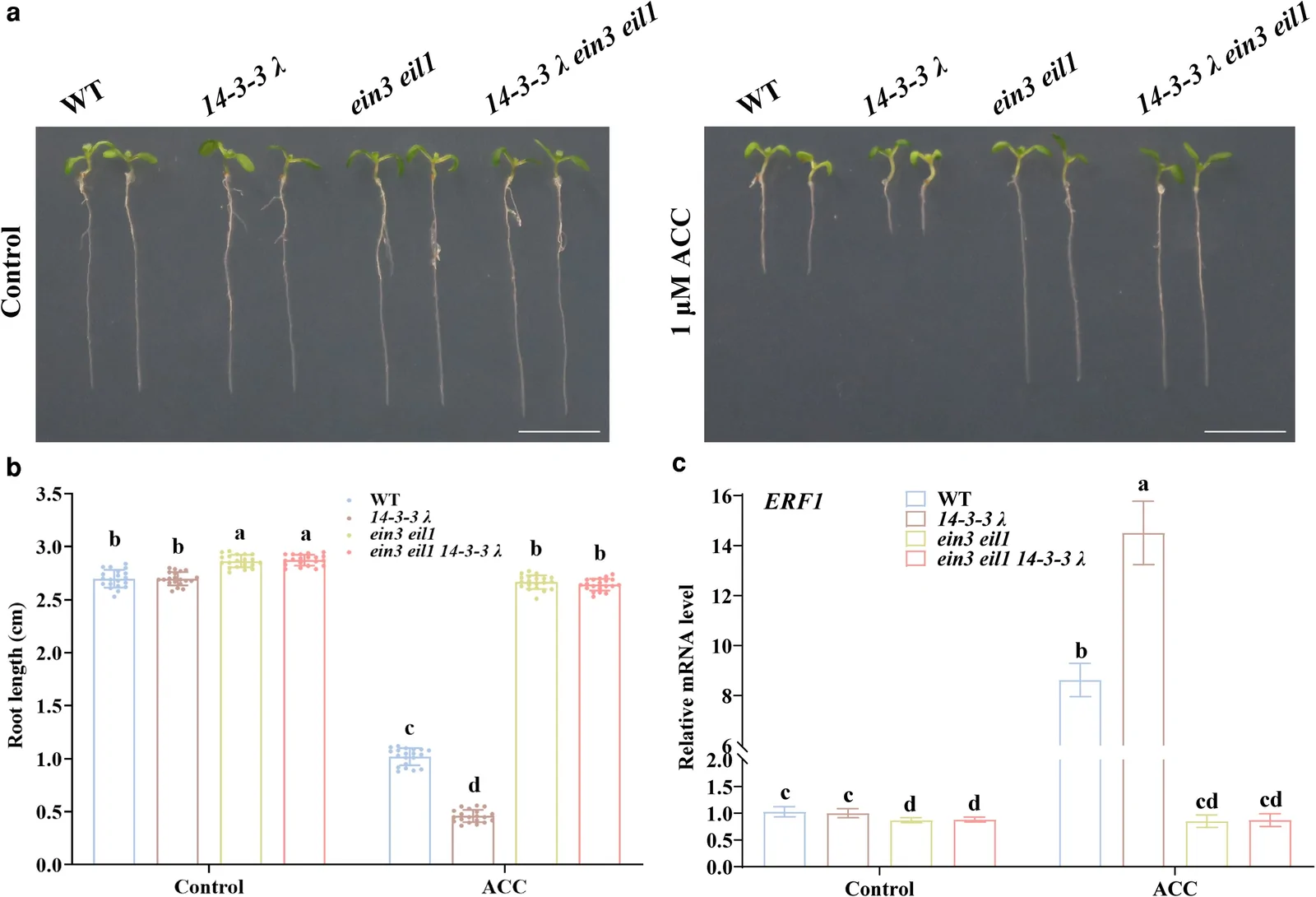
Genetic interaction of *14-3-3 λ* and *EIN3/EIL1* genes. (a) The primary root elongation phenotype of WT, *14-3-3 λ*, *ein3 eil1*, and *14-3-3 λ ein3 eil1*. Seedlings were grown on 1/2 MS medium supplemented without or with 1 μM ACC for 7 days. Scale bar = 1 cm. (b) Quantitative analysis of primary root length in WT, *14-3-3 λ*, *ein3 eil1*, and *14-3-3 λ ein3 eil1*. Root lengths were measured 7 days after germination on 1/2 MS medium supplemented with or without 1 μM ACC. Data represent mean ± SD (n = 20). (c) Relative expression levels of *ERF1* were analyzed by RT-qPCR in WT, *14-3-3 λ*, *ein3 eil1*, and *14-3-3 λ ein3 eil1*. Data are presented as means ± SD from 3 independent biological replicates. Different letters indicate statistically significant differences (*P* < 0.05) as determined by 1-way ANOVA followed by Tukey's multiple comparison test.

Building on the above findings, we further examined the genetic relationship between EIN3/EIL1 and 14-3-3 λ in regulating ethylene-inhibited primary root growth. A higher-order mutant, *14-3-3 λ ein3 eil1*, was generated through genetic crossing. As expected, the *ein3 eil1* mutant showed strong insensitivity to ACC (Fig. 7a and b), consistent with its lack of response to ethylene signaling (An et al. 2010). The *14-3-3 λ* mutant was hypersensitive to ACC, while the *14-3-3 λ ein3 eil1* mutant showed identical ACC sensitivity to *ein3 eil1* in terms of primary root length and *ERF1* expression levels upon ACC treatment (Fig. 7a-c). These results indicate that EIN3/EIL1 are epistatic to 14-3-3 λ in mediating the ethylene-induced inhibition of primary root growth.

## Discussion

In recent years, agricultural production has been significantly threatened due to the reduction in arable land area, decline in soil quality, abnormal climate, and scarcity of water resources. Immobile by nature, plants possess neither locomotion nor a nervous system; rooted in place, they must endure every environmental insult. To ensure better survival and reproduction, plants must possess a series of abilities to withstand environmental stresses. Plant root not only anchors the above-ground parts of the plant in the soil and absorbs and transports water and nutrients from the soil but also perceives internal and external stimuli, integrating these signals to regulate their own life activities most advantageously to adapt to the environment. Therefore, studying the root growth process of plants is beneficial for enhancing their stress resistance, providing a solid foundation for increasing crop yield and quality.

Among the many endogenous signals that shape root architecture, the gaseous hormone ethylene has emerged as a particularly well-studied regulator (Qin and Huang 2018; Qin et al. 2020; Khoury et al. 2024). Our study adds a layer to this regulatory network by identifying 14-3-3 λ as a direct, post-translational modulator of the core ethylene signaling pathway. Specifically, we demonstrate that 14-3-3 λ physically interacts with EIN3/EIL1, attenuates their binding to the *ERF1* promoter, and thereby represses ethylene-induced primary root elongation (Fig. 1 and Fig. 5). Consistent with this, we observed that *14-3-3 λ* displays significantly shorter primary roots than WT under ACC treatment. One of the most widely documented ethylene responses is the triple response of etiolated seedlings. For instance, in the presence of ethylene or ACC, dark-grown Arabidopsis thaliana seedlings develop a short, thickened root and hypocotyl with exaggerated curvature of the apical hook (Ecker 1995; Roman and Ecker 1995). We further investigated the triple response phenotype of 14-3-3 λ, but no significant differences were observed compared to the WT. Therefore, the increased sensitivity of the *14-3-3 λ* to ethylene in primary root growth may represent a tissue-specific ethylene response. As reported previously, ethylene responses are inherently tissue-specific, with distinct regulatory modules governing outputs in different organs (Stepanova et al. 2008; Zander et al. 2014; Wang et al. 2020). For instance, *taa1* mutants exhibit ethylene insensitivity in primary root elongation but retain a normal triple response in hypocotyls and apical hooks (Stepanova et al. 2008). Similarly, class II TGA transcription factors are essential for ethylene-induced defense gene expression in leaves and the activation of *PDF1.2* (a marker of jasmonate-ethylene crosstalk) in roots, yet their loss does not alter the triple response of etiolated seedlings (Zander et al. 2014). In addition, TREE1, a transcriptional repressor interacting with EIN3, specifically mediates ethylene-induced shoot growth inhibition but has no impact on root ethylene responses (Wang et al. 2020). Our findings, in conjunction with existing literature, confirm that beyond the classical triple response, tissue-specific regulation constitutes another crucial component in ethylene signaling. Upon ACC perception, the repressive interaction between 14-3-3 λ and EIN3/EIL1 is relieved, allowing EIN3/EIL1 to activate downstream targets. This mechanism positions 14-3-3 λ as a tunable brake that fine-tunes root growth under basal conditions, while enabling rapid ethylene response activation when needed. Collectively, our findings highlight that ethylene signaling employs tissue-specific modulators to tailor responses across organs. The distinct phenotypes of *14-3-3 λ* mutants in roots versus etiolated seedlings reinforce this concept, adding a layer to our understanding of the complex ethylene regulatory network.

14-3-3 proteins are conserved phosphopeptide-binding factors in eukaryotes, playing key roles in plant growth, development, and responses to biotic/abiotic stresses (Cotelle et al. 2000; Denison et al. 2011; Wang et al. 2011). Typically, they interact with phosphorylated proteins to modulate signal transduction by altering activity, stability, conformation, subcellular localization of target proteins, or binding affinity for other factors (Paul et al. 2012; de Boer et al. 2013). The 14-3-3 proteins have 3 high-affinity phosphoserine motifs: RSXpS/TXP (mode 1), RXXXpS/TXP (mode 2), and pS/T-X(1-2)-COOH (mode 3) (Yaffe et al. 1997). Unfortunately, we did not find any canonical binding motifs in the EIN3. To verify if EIN3 phosphorylation underpins its interaction with 14-3-3 λ, we used a pan-phospho-serine/threonine antibody (anti-pSer/Thr) to detect EIN3 phosphorylation status with/without ACC treatment. No signals were observed in either group (Figure S6a), indicating that EIN3 is not phosphorylated under these conditions. This was further confirmed by mass spectrometry analysis of phosphorylation modifications (Figure S6b and c). Emerging research indicates that 14-3-3 proteins can also bind to atypical motifs of certain proteins and are even capable of binding to non-phosphorylated motifs (de Boer et al. 2013; Liu et al. 2017). In animals, the majority of 14-3-3 protein-binding motifs are located within disordered regions. The disorder enhances molecular recognition and protein-protein interactions, thereby facilitating the interaction of proteins with multiple partners (Bustos 2012). By using PLAAC (http://plaac.wi.mit.edu/), we examined the structure of the EIN3 protein and found that the C-terminus of EIN3 is disordered (Figure S6d), indicating that the 14-3-3 λ protein may interact with the EIN3 protein through this region. To verify this hypothesis, we performed yeast 2-hybrid analysis by truncating the C-terminus of EIN3 protein and testing its interaction with 14-3-3 λ. The results showed that the interaction between EIN3 and 14-3-3 λ disappeared when the C-terminus containing the disordered region was removed, indicating that the C-terminus of EIN3 is essential for their interaction (Figure S6e and f). Previous work demonstrated isoform-specific control of ethylene biosynthesis: 14-3-3 ψ destabilizes ACS5/ACS9 to decrease ethylene production, whereas 14-3-3 ω stabilizes ACS2/ACS6 to increase it (Yoon and Kieber 2013; Catala et al. 2014). Extending this paradigm to transcriptional modulation, our findings show that 14-3-3 proteins not only regulate ACS turnover but also gate the activity of EIN3/EIL1. Mechanistically, the ACC-induced dissociation of 14-3-3 λ from EIN3/EIL1 is reminiscent of other hormone-dependent releases of 14-3-3, such as the auxin-triggered liberation of ARF activators from AUX/IAA repressors (Weijers and Wagner 2016). Whether ACC promotes ubiquitination, or conformational changes that weaken the interface of 14-3-3 λ and EIN3/EIL1 remains to be determined.

The convergence of 14-3-3, *EIN3/EIL1*, and *ERF1* places ethylene signaling at the nexus of multiple hormonal pathways. ERF1 itself integrates JA and ethylene inputs to regulate defense genes (Lorenzo et al. 2003), while ERF109 modulates auxin biosynthesis in response to wounding (Zhang et al. 2019b). Thus, 14-3-3 λ may act as a molecular “hub” that translates not only ethylene levels but also cross-talk with JA, auxin, and possibly ABA into coherent root growth outputs. Dissecting these higher-order interactions will be essential for predictive engineering of root architecture under climate change.

**Figure 8:**
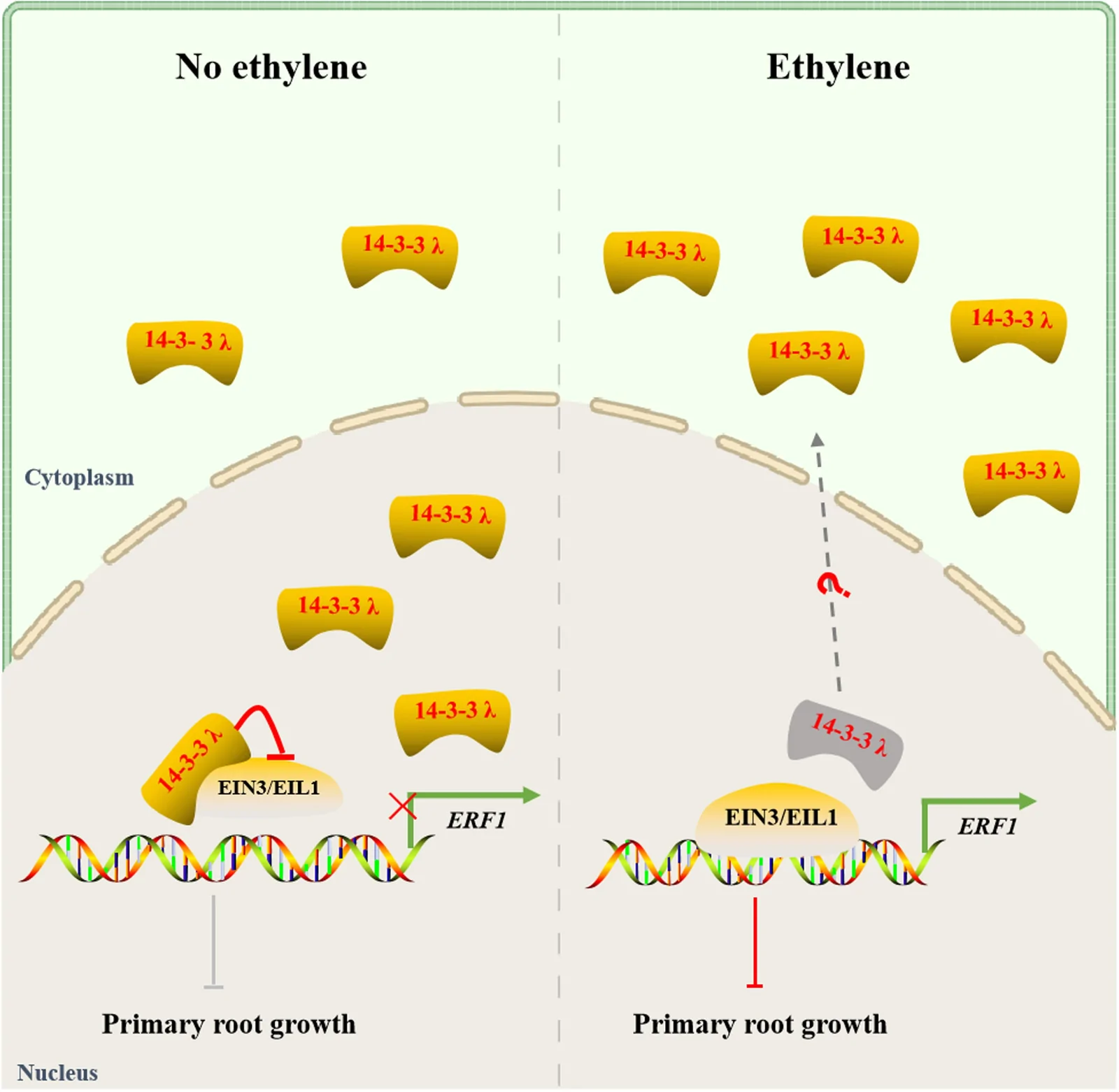
A working model illustrating the regulatory role of 14-3-3 λ in ethylene-mediated primary root growth inhibition. In the absence of ethylene, the nuclear 14-3-3 λ protein interacts with EIN3/EIL1, suppressing both its transcriptional activation activity and binding capacity to *ERF1*. Upon ACC treatment, 14-3-3 λ may translocate out of the nucleus through an unknown pathway, which reduces its interaction with EIN3/EIL1. This release of repression enables EIN3/EIL1 to bind to *ERF1* and activate its expression, consequently inhibiting primary root growth. 14-3-3 proteins function as modulators to maintain the basal inactivation of the ethylene signaling pathway in the absence of ACC, ensuring proper root growth homeostasis.

In conclusion, our results demonstrate the interaction between 14-3-3 λ and EIN3/EIL1, thus interfering with the transcriptional activation effects of EIN3/EIL1 on the downstream target gene *ERF1* under normal condition, which inhibits the ethylene signaling transducer. However, exogenous ACC treatment can weaken the interaction between 14-3-3 λ and EIN3/EIL1 by promoting the cytoplasmic localization of 14-3-3 λ, thereby restoring the transcriptional activity of EIN3/EIL1 and activating ethylene signaling to inhibit primary root growth (Fig. 8). By integrating post-translational regulation with transcriptional output, this mechanism highlights how 14-3-3 proteins serve as adjustable checkpoints within the complex plant hormone signaling network and provides a molecular framework for engineering root systems to enhance stress resistance.

## Materials and methods

### Plant materials and growth conditions

*Arabidopsis* (*Arabidopsis thaliana*) ecotype Columbia-0 (Col-0) was used as wild-type (WT) in this study. *Arabidopsis thaliana 14-3-3 κ* (T-DNA mutant, SALK_071097), *14-3-3 λ* (T-DNA mutant, SALK_075219), *14-3-3 κ λ* double mutant, *35S::14-3-3 λ* (*#2,* #*4,* and *#7*) transgenic lines (Zhang et al. 2019a), *ein3-1* (Li et al. 2015), and the *EBS::GUS* reporter line (Li et al. 2015) were lab stock. *ein3-1 eil1-1* (Alonso et al. 2003) have been described elsewhere. *14-3-3 λ ein3 eil1* and *EBS::GUS 14-3-3 λ* were obtained by genetic crossing. We generated the complementary lines (*COM #1, #6,* and *#9*) by expressing the full-length coding sequence of *14-3-3 λ* driven under its native promoter in *14-3-3 λ* mutant. *35S::EIN3-FLAG ein3-1* transgenic plants were obtained by cloning *EIN3-FLAG* coding sequences into the pCAMBIA1300s vector and transforming the constructs into the *ein3-1* background. Seeds were surface sterilized with 10% (w/v) sodium hypochlorite (NaClO) for 5 minutes, followed by 5 washes with sterile water. These seeds were placed in the dark at 4℃ for 2 days. Subsequently, the seeds were sown in half MS medium (pH 5.8, containing 1% [w/v] sucrose and 0.8% [w/v] agar), and then the seeds were germinated and grown under a 16:8 h light:dark photoperiod at 100 μmol m^−2^ s^−1^ at 22 °C for 8 days. Primer sequences are listed in Table S1.

### Bioinformatics analyses

The 3D structure of the 14-3-3 λ-EIN3/EIL1 interaction was predicted and modeled using the Alphafold server (Abramson et al. 2024). Protein-protein docking of the obtained proteins was performed using GRAMM (https://gramm.compbio.ku.edu/). The chemical bonds of the results were analyzed using Ligplot^+^ software, and visualization was carried out using Pymol software.

### Identification and phylogenetic analysis of 14-3-3 λ family members

To identify putative homologs of 14-3-3 λ (AT5G10450), the protein sequences were queried against the Conserved Domain Database (CDD) at NCBI (https://www.ncbi.nlm.nih.gov/Structure/cdd/wrpsb.cgi). The amino acid sequences corresponding to the identified conserved domains were then utilized as seed sequences for HMMER-based searches within Phytozome 13, encompassing multiple species, including *Chlamydomonas reinhardtii* (version 6.1), *Physcomitrium patens* (version 6.1), *Selaginella moellendorffii* (version 1.0), *Thuja plicata* (version 3.1c), *Arabidopsis thaliana* (version TAIR10), *Solanum lycopersicum* (version ITAG4.0), *Oryza sativa* (version 7.0), *Glycine max* (version Wm82.a6.v1), and *Zea mays* (version B73-REFERENCE-NAM-5.0). Phylogenetic relationships were inferred using MEGA 11 (Molecular Evolutionary Genetics Analysis, version 11) (Tamura et al. 2021). Sequence alignment was performed using the ClustalW method with default settings, and the resulting alignment was used to construct a phylogenetic tree employing the neighbor-joining method with 1,000 bootstrap replicates.

### Protein-protein interaction

Y2H experiments were conducted using the Matchmaker ^TM^ Gold Yeast Two-Hybrid System according to the manufacturer (Clontech). The CDS of *14-3-3 λ* was fused to the DNA binding domain (BD) in *pGBKT7,* and the CDS of *EIN3/EIL1* were individually cloned into *pGADT7*. Yeast transformants were selected on double dropout medium lacking Trp and Leu (SD-WL), and protein interactions were analyzed on quadruple dropout medium lacking Trp, Leu, His, and Ade (SD-WLHA). For BiFC analysis, the CDS of *14-3-3 λ* was cloned into *pSPYNE* containing the N-terminal of YFP (YFP^N^-14-3-3 λ), and the CDS of *EIN3/EIL1* was cloned into *pSPYCE* containing the C-terminal of YFP (YFP^C^-EIN3/EIL1). YFP^N^-14-3-3 λ and YFP^C^-EIN3/EIL1 or YFP^N^-14-3-3 λ and YFP^C^ were co-expressed in *Nicotiana benthamiana (N. benthamiana)* leaves for 2 days, after which YFP (excitation 488 nm and emission 520–560 nm) fluorescence signals were detected using an LSM 980 laser scanning confocal microscope (Carl Zeiss). For Co-IP assays, the CDS of *EIN3* and *EIL1* were cloned into *pCAMBIA1300*, including sequences encoding a GFP tag fused to its C-terminus and under the control of the *35S* promoter. EIN3/EIL1-GFP and 14-3-3 λ-FLAG were transformed into *Agrobacterium* GV3101 and infiltrated into leaves of 4-week-old *N. benthamiana.* After 2 days, total proteins were extracted from the infected leaves by homogenization in IP buffer (150 mM NaCl, 25 mM Tris-HCl, pH 7.5, 0.2% NP-40, 1 mM phenylmethylsulfonyl fluoride, and 1× protease inhibitor cocktail) and then immunoprecipitated by anti-GFP antibody. The resulting precipitates were resuspended and detected using anti-GFP and anti-FLAG antibodies, respectively.

### Gene expression analysis

The procedure has been described previously (Ding et al., 2022b). Briefly, the roots were collected for total RNA isolation using TRIzol Reagent (TransGen Biotech), and the cDNA was synthesized by reverse transcription after the removal of DNA contamination (TransGen Biotech). The qPCR was performed in 96-well plates with SYBR Green I dye (Monad) and then placed on a Bio-Rad CFX96 apparatus under the following conditions: 95℃/3 min for pre-denaturation, followed by 95℃/15 s and 60℃/30 s for 45 cycles. *ACT2/8* were chosen as internal reference genes. All these experiments were biologically duplicated at least 3 times. The primer sequences are listed in Table S1.

### Measurement of ethylene production

Ethylene measurement was conducted according to the previously described methods (Hansen et al. 2009; Sun et al. 2017). Whole-root samples (200 mg) were excised and placed into an 8 mL airtight vial containing 2 mL of agar medium (1% [m/v] agar); 1 mL sample was taken from the headspace of the vial and injected into a gas chromatograph equipped with a flame ionization detector column, which was packed with aluminum activated at 100 °C. Ethylene was detected by the ionization detector and recorded by an integrator. The sample injection temperature was 80 °C, while the column temperature was 150 °C

### Preparation of anti-14-3-3 λ polyclonal antibodies

Recombinant polyhistidine-tagged 14-3-3 λ prepared from *E. coli* was used as an antigen for polyclonal antibody production in rabbits. 2 mg of puriﬁed recombinant protein was used for rabbit immunization. The post-immunization sera were utilized for antibody specificity testing, with samples derived from 7-day-old WT, *14-3-3 λ* mutant, *14-3-3 κ* mutant, and *14-3-3 κ λ* double mutant.

### Western blot

As previously described (Ding et al., 2022b), total proteins were extracted using a plant protein extraction buffer containing 375 mM NaCl, 2.5 mM EDTA, 1% β-mercaptoethanol, 125 mM Tris-HCl (pH 8.0), and 1% SDS. Subsequently, the proteins were separated by electrophoresis using a 12% SDS-PAGE gel. Immunoblotting was performed on PVDF membranes following standard procedures. The anti-14-3-3 λ, anti-EIN3 (Guo and Ecker 2003; Zhang et al. 2014), anti-FLAG (Beijing Solarbio Science & Technology Co., Ltd. K200001M), anti-GFP, and anti-ACTIN (Orizymes Biotechnologies (Shanghai) PAB220836, PAB001C) antibodies were diluted 1,000-fold with TBST buffer (20 mM Tris-HCl, pH 7.5, 150 mM NaCl, and 1% Tween 20) for incubation with membranes.

### Dual-luciferase reporter assay

Constructs of *14-3-3 λ*, *EIN3,* and *EIL1* were used as effectors; the *ERF1* promoter was cloned into *pGreen0800*, and the LUC expression was controlled by *ERF1* promoter activity. The resulting reporters were transiently co-expressed with or without the effectors above in 4-week-old *N. benthamiana* leaves for 2 days, and then the activities of LUC and REN were detected by previously reported methods (Guo et al. 2022a, 2022b; Liu et al. 2022).

### Pull-down assays

The total protein of *35S::EIN3-FLAG ein3-1* was enriched by the pull-down method, and the supernatant was incubated overnight with anti-FLAG affinity gel beads (Sigma-Aldrich) at 4 °C with gentle shaking. The beads were washed 3 times at 1,000 g centrifugation for 1 min and washed with washing buffer (50 mM HEPES-KOH, pH 7.0, 150 mM NaCl, 0.5% dodecyl maltoside, and a protease inhibitor cocktail tablet). Subsequently, the immunoprecipitate was subjected to boiling and elution using 1× SDS-PAGE sample buffer, followed by utilization in SDS-PAGE protein separation and western blot analysis by anti-14-3-3 λ antibody.

### GUS staining and GUS activity

GUS staining was used as described previously (Ding et al., 2022a). Untreated and ACC-treated *EBS::GUS* and *EBS::GUS 14-3-3 λ* seedlings were stained in GUS staining solution (100 mM sodium phosphate buffer, pH 7.5, 10 mM EDTA, 0.5 mM potassium ferricyanide, 0.5 mM potassium ferrocyanide, 1 mM 5-bromochloro-3-indolyl-b-D-glucuronide, and 0.1% Triton X-100). The samples were rinsed with 70% ethanol several times to remove the chlorophyll, and images were then taken under a microscope. Relative GUS activities of *EBS::GUS* and *EBS::GUS 14-3-3 λ* seedlings were assayed according to a method described previously.

### ChIP-qPCR analysis

ChIP analysis according to a previously reported method (Fu et al. 2021). In brief, chromatin was isolated from 7-day-old *35S::14-3-3 λ* seedlings treated with or without 10 μM ACC for 12 h. The anti-14-3-3 λ was used for protein immunoprecipitation. DNA fragments in both input and immunoprecipitated samples were quantified by qPCR. The *ACTIN7* was used as a reference gene. At least 3 independent experiments were performed. The primers used for ChIP-qPCR are listed in Table S1.

### *In vivo* phosphorylation assay

Phosphorylation assay according to the previously reported method (Wang et al. 2022). In brief, 7-day-old *35S::EIN3-FLAG/ein3 eil1* transgenic seedlings were treated with or without 10 μM ACC for 12 h, followed by total protein extraction in a buffer containing protease and phosphatase inhibitors. EIN3-FLAG was enriched via immunoprecipitation (IP) with FLAG-tag beads, and eluted proteins were standardized to equal concentrations using a BCA assay. Phosphorylation was detected by immunoblotting with a pan phospho-serine/threonine antibody (ABclonal, AP0893) after SDS-PAGE separation.

### PLAAC

PLAAC (http://plaac.wi.mit.edu/) is a web application that scans protein sequences for domains with prion-like amino acid composition (most notably enriched for Q or N) based on a hidden-Markov model (HMM) algorithm. Prion-like domain (PrLD) scores generated by PLAAC range from 0 to 1—a score of 0 indicates the absence of a PrLD, while a score greater than 0.5 suggests a high likelihood of containing a PrLD (with scores closer to 1 reflecting increased probability). PLAAC offers a convenient PrLD prediction for target proteins. Users can either upload sequence files in FASTA format or paste sequences directly into the input textbox. The tool then ranks input sequences based on multiple summary scores and visualizes these scores along the length of the sequences. For PrLD prediction in this study, PLAAC was used with default parameters, except for the relative weighting of background probabilities, which was set to *A. thaliana*.

### Accession numbers

Accession numbers of major genes/proteins mentioned in this paper: *EIN3* (AT3G20770); *EIL1* (AT2G27050); *14-3-3 λ* (AT5G10450); *14-3-3 κ* (AT5G65430); *ERF1* (AT3G23240); *ACS2* (AT1G01480); *ACS5* (AT5G65800); *ACS6* (AT4G11280); *ACS8* (AT4G37770); *ACO1* (AT2G19590); *ACO2* (AT1G62380); *ACO4* (AT1G05010); *CTR1* (AT5G03730); *EIN2* (AT5G03280)

## Supplementary Material

kiag229_Supplementary_Data

## Data Availability

The data underlying this article are available in the article and in its online supplementary material.
