# Correction: 14-3-3 λ suppresses ethylene-mediated root growth inhibition through EIN3/EIL1 in Arabidopsis

**DOI:** 10.1093/plphys/kiag685

**Published:** 2026-09-22

This is a correction to: Zhi-Xin Xiang, Yong-Lun Lv, Ying-Rui Li, Yi-Feng Zhou, Yan-Ke Lu, Meng Xu, Feng Ding, 14-3-3 λ suppresses ethylene-mediated root growth inhibition through EIN3/EIL1 in Arabidopsis, *Plant Physiology*, Volume 201, Issue 1, May 2026, kiag229, https://doi.org/10.1093/plphys/kiag229

In the originally published version of the paper there was a typographical error in the title. The error in the article, “Aratbidopsis”, has been corrected to read: “Arabidopsis”.

