# Supplementary material for: 14-3-3 λ suppresses ethylene-mediated root growth inhibition through EIN3/EIL1 in Aratbidopsis
Source: Plant Physiol. 2026 Apr 21;201(1):kiag229. doi: 10.1093/plphys/kiag229 (PMC13222030; doi:10.1093/plphys/kiag229)
Supplement: kiag229_Supplementary_Data [file kiag229_supplementary_data.pdf]

Supplemental Figures

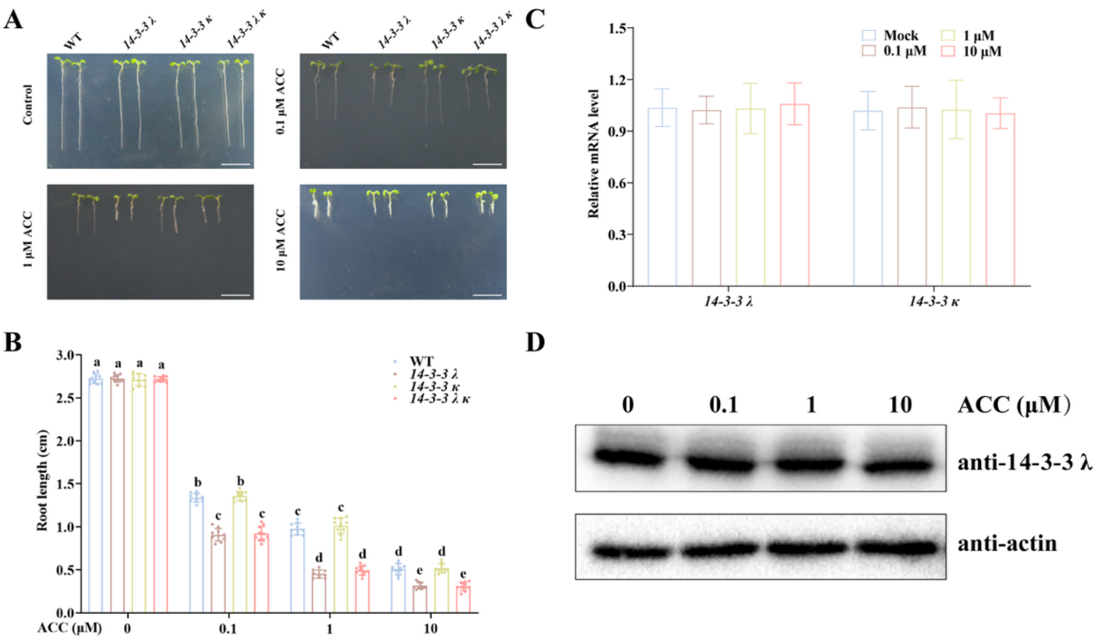

Supplementary Figure S1. 14-3-3 λ rather than 14-3-3 κ is involved in ethylene-mediated inhibition of primary root growth. (A) The primary root elongation phenotype of the wild type (WT), *14-3-3 λ*, *14-3-3 κ*, and *14-3-3 κ λ*. Seedlings were grown on 1/2 MS medium supplemented with 0, 0.1, 1, or 10 µM ACC for 7 days. Scale bars = 1 cm. **(B)** Quantitative analysis of primary root length in the WT, *14-3-3 λ*, *14-3-3 κ*, and *14-3-3 κ λ*. Data represent mean ± SD (n = 12). **(C)** Transcript levels of *14-3-3 λ* and *14-3-3 κ* in the WT. **(D)** The accumulation of 14-3-3 λ protein in 7-day-old wild-type seedlings treated with 0, 0.1, 1, or 10 µM ACC was immunodetected using an anti-14-3-3 λ antibody. Anti-actin was used as a loading control. Data are presented as means ± SD from three independent biological replicates. Different letters indicate statistically significant differences ($P < 0.05$) as determined by one-way ANOVA followed by Tukey's multiple comparison test.

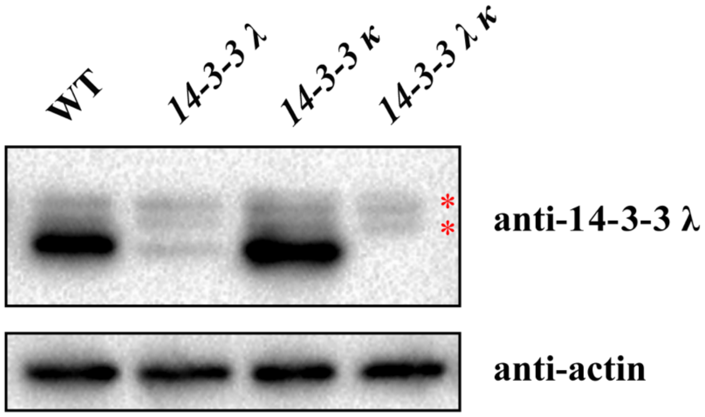

Supplementary Figure S2. The levels of 14-3-3 λ protein were analyzed in the WT, *14-3-3 λ*, *14-3-3 κ*, and 1*4-3-3 κ λ*. *, nonspecific band. Anti-actin served as a loading control.

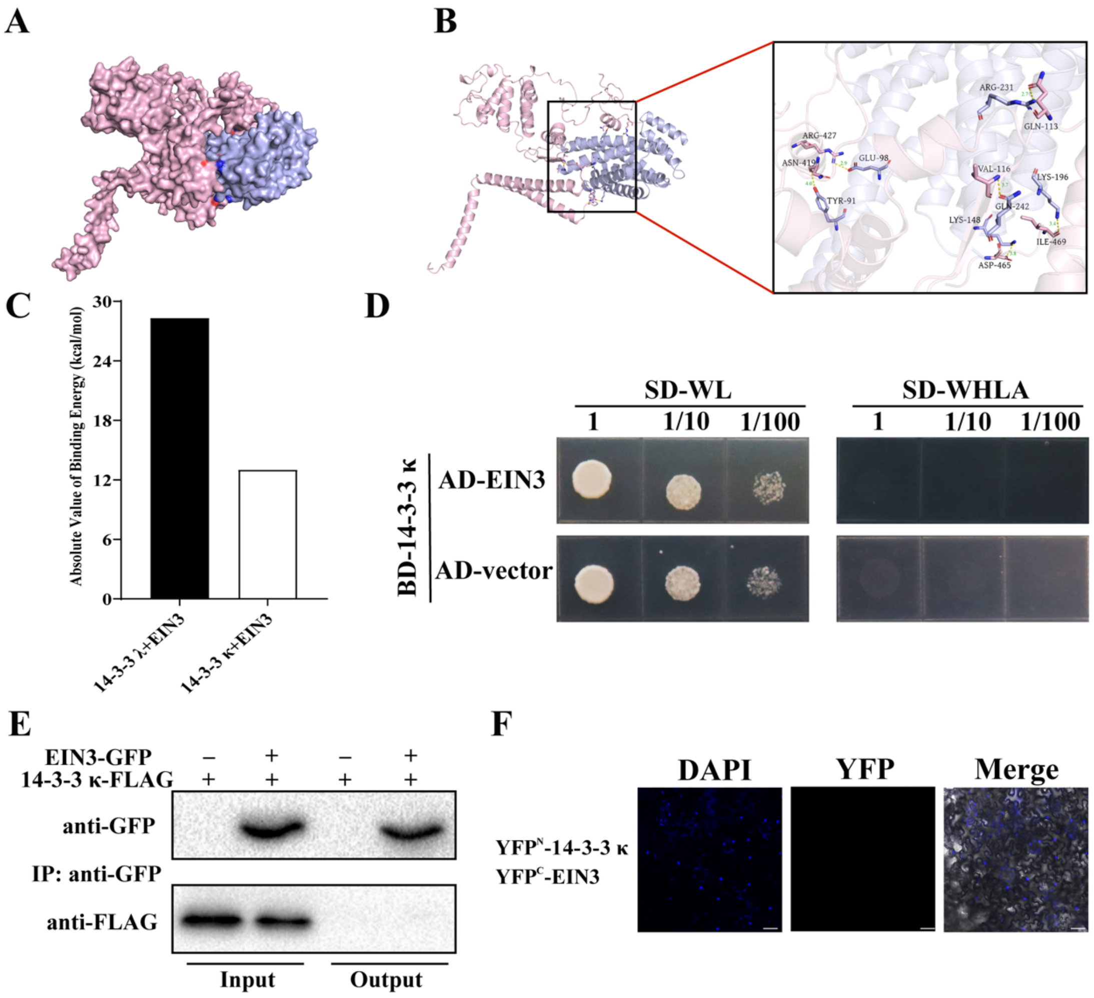

Supplementary Figure S3. 14-3-3 κ does not interact with EIN3. (**A**) Structural model of the 14-3-3 κ/EIN3 complex predicted by AlphaFold. EIN3 is shown in wine red and 14-3-3 κ in blue-purple. (**B**) Close-up view of the main interaction interface between 14-3-3 κ and EIN3, highlighting specific molecular interactions predicted by AlphaFold. EIN3 is shown in wine red and 14-3-3 κ in blue-purple. Key residues at the binding interface are displayed in stick representation (right). (**C**) Quantification of the absolute binding energy between 14-3-3 λ/14-3-3 κ and EIN3. Values are presented as the absolute value of binding energy (kcal/mol). **(D)** Yeast two-hybrid. **(E)** Co-IP analysis. **(F)** BiFC analysis.

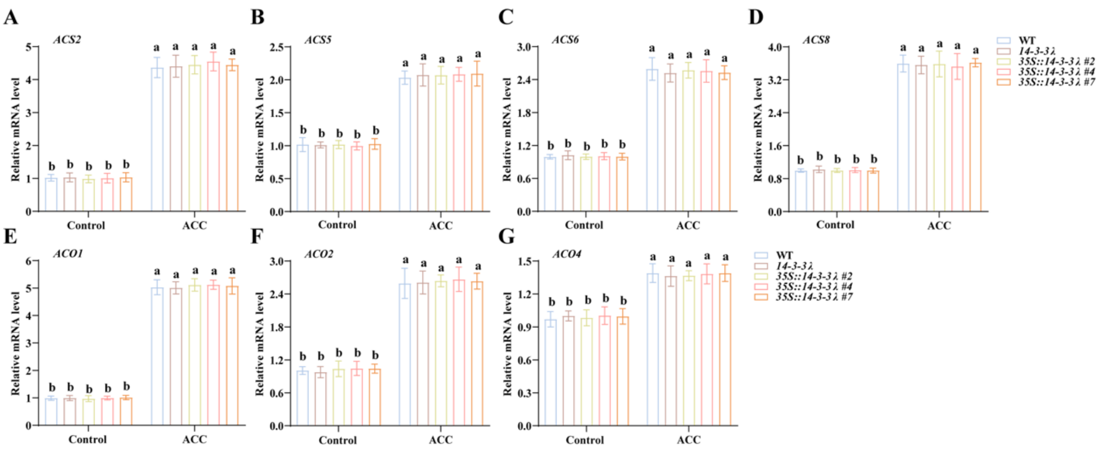

Supplementary Figure S4. 14-3-3 λ does not affect expression of ethylene biosynthesis–related genes. **(A–G)** RT-qPCR analysis of *ACS2*, *ACS5*, *ACS6*, *ACS8*, *ACO1*, *ACO2*, and *ACO4* expression in 7-d-old WT, *14-3-3 λ*, and *35S:14-3-3 λ* (*#2*, *#4*, *#7*) seedlings treated or not with 1 μM ACC for 24 h. Data are presented as means ± SD from three independent biological replicates. Different letters indicate statistically significant differences (*P* < 0.05) as determined by one-way ANOVA followed by Tukey's multiple comparison test

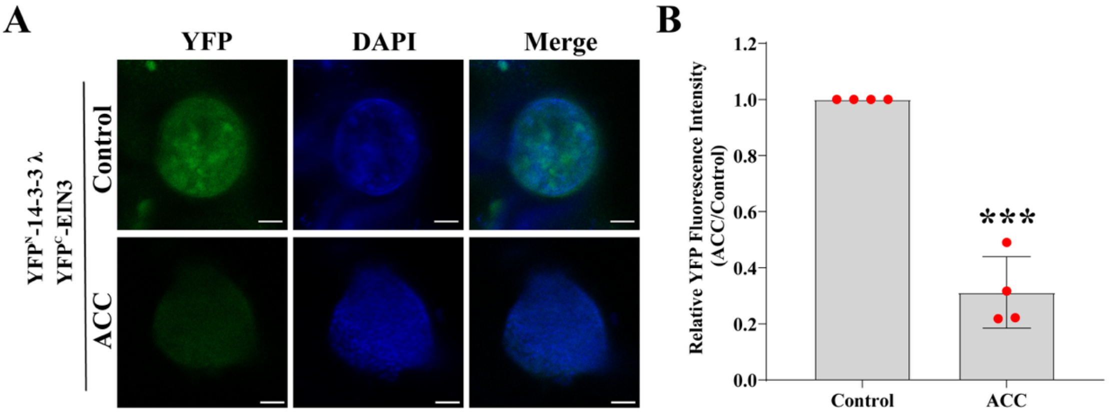

Supplementary Figure S5. ACC reduces the interaction between 14-3-3 λ and EIN3. (**A**) BiFC analysis of the interaction between 14-3-3 λ and EIN3 in *Nicotiana benthamiana* leaf epidermal cells under control and ACC treatment conditions. Bars = 5 μm. (**B**) Quantification of relative YFP fluorescence intensity in nuclei from (**A**), normalized to the control. Asterisks indicate significant differences between the indicated columns (Student's *t*-test): ***, *P* < 0.001. Data represent mean ± SD (n = 4).

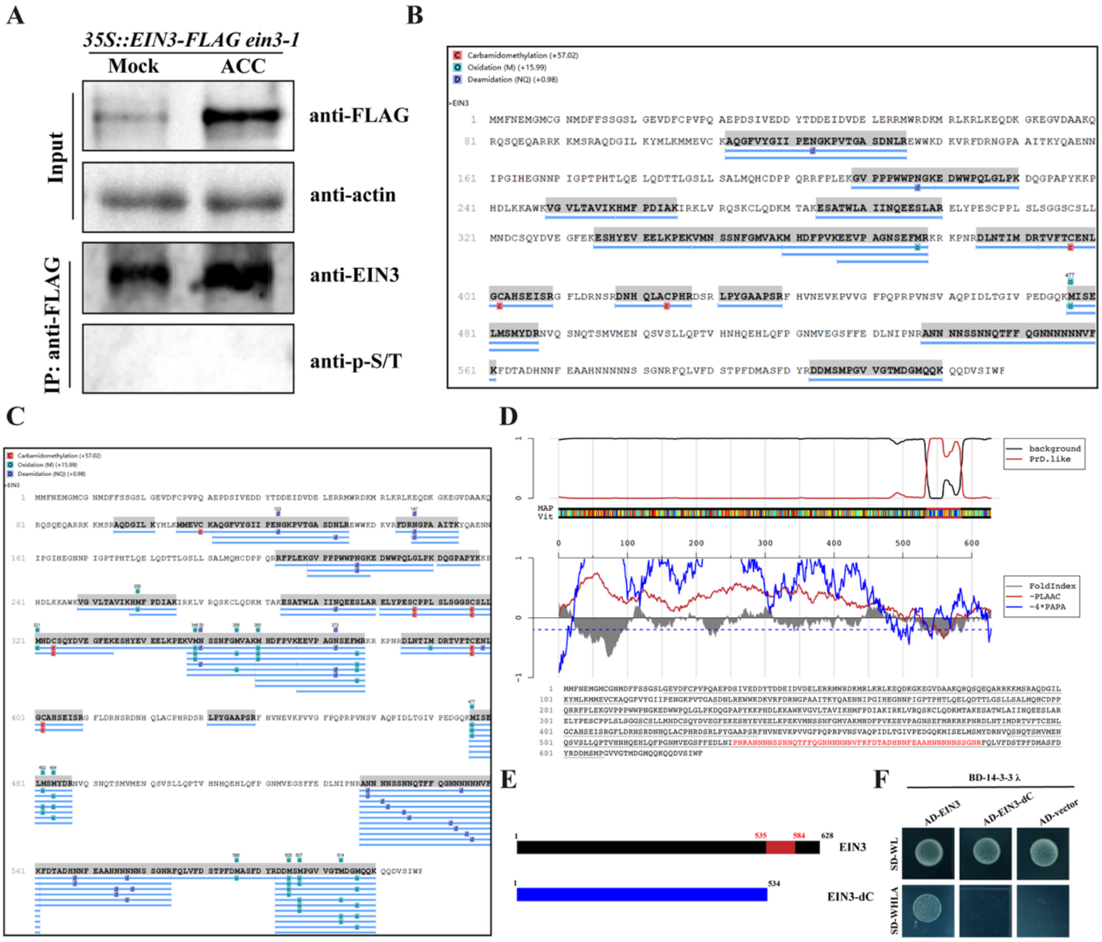

Supplementary Figure S6. Detection of EIN3 phosphorylation status. (**A**) Immunoblot analysis of EIN3-FLAG protein levels (Input, anti-FLAG) and its phosphorylation status (IP: anti-FLAG, followed by anti-phospho-Ser/Thr [anti-p-S/T]) in *35S:EIN3-FLAG ein3-1* seedlings under mock (no ACC) and ACC treatment conditions. Anti-actin serves as a loading control. (**B** and **C**) Mass spectrometry-identified post-translational modification (PTM) site mapping of EIN3 in *35S:EIN3-FLAG ein3-1* seedlings under mock (**B**) and ACC treatment (**C**) conditions. (**D**) Prediction of intrinsically disordered regions (IDRs) and prion-like domains (PrLDs) in EIN3 protein by 'Prion-like Amino Acid Composition' (PLAAC; http://plaac.wi.mit.edu/). (Top) PrLDs prediction of EIN3 (red curve: threshold set to 0, black curve: background control). (Middle) EIN3 disordered region prediction (red curve: regions below the dashed line correspond to disordered regions) and IUPred3 (blue curve: higher values reflect greater disorder tendency). (Bottom) EIN3 amino acid sequence, with FoldIndex-predicted disordered regions shaded gray. (**E**) Schematic diagram of EIN3 truncation for yeast two-hybrid assay. The red portion corresponds to the disordered region of EIN3, and EIN3-dC represents the C-terminal truncated fragment of the EIN3 protein. (**F**) Yeast two-hybrid assay.

Supplemental Table 1. List of all primers used in this study.

**Primer name**	**Sequence (5'-3')**
**Genomic DNA PCR**	
SALK_075219 LP	TAAGGCTTGAGCAGAGTCGAG
SALK_075219 RP	TTCTTTCTTTTTCGCTGCTTG
SALK_071097 LP	TCGAGGAACTCACAAACCAAG
SALK_071097 RP	AGGCTTGGTTTGGCTCTTAAC
LBb1.3	ATTTTGCCGATTTCGGAAC
**RT-qPCR**	
ACS2 RT-F	TCATGGGAAAAGCTAGAGGTGGAAG
ACS2 RT-R	TCAACGGTTAATTTGAAATTGTCGG
ACS5 RT-F	TCGACATCTGCGAATGAGACT
ACS5 RT-R	TCTCCATTTAAGATCTCTATCAAATCC
ACS6 RT-F	AAACCGATGGCTGCAACAACTATGAT
ACS6 RT-R	TAAGTCTGTGCACGGACTAGCGGAG
ACS8 RT-F	TGGGGTGATTTACTCCAACGATGATT
ACS8 RT-R	GACACTCGATGCCTGCAGCCTCTAG
AC01 RT-F	AGAGAGATGGAGATTCCAGTTATTG
AC01 RT-R	GAGTGAAGTAGTATAGCTGAATCAG
AC02 RT-F	GATGAATACAGGACGGCCATGAAAGAC
AC02 RT-R	CATCTTCCATAAACATAAACACCCCACAC
AC04 RT-F	CTCGACGACGATTACAGAACGTTAATG
AC04 RT-R	GAACTCTACGAACAGAAAACATTAATTCCC
ERF1 RT-F	ATTCTTTCTCATCCTCTTCTTCT
ERF1 RT-R	CGAATCTCTTATCTCCGCCG
ACTIN2 RT-F	CTTGCACCAAGCAGCATGAA
ACTIN2 RT-R	CCGATCCAGACACTGTACTTCCTT
**Molecular Cloning**	
pGAD-*EIN3* F	GGCATCGATACGGGATCCATGATGTTTAATGAGATGGG
pGAD-*EIN3* R	CTCGAGCTCGATGGATCCGAACCATATGGATACATCTT
pGAD-*EIL1* F	GGCATCGATACGGGATCCATGATGATGTTTAACGAGAT
pGAD-*EIL1* R	CTCGAGCTCGATGGATCCTCAGAACCATATTGATACAT
pGBD -*14-3-3 λ* F	CCGAATTCCCGGGGATCCATGGCGGCGACATTAGGCAG
pGBD -*14-3-3 λ* R	CTGCAGGTCGACGGATCCTTACATAGAGTAGTAATAAC
pGBD -*14-3-3 κ* F	CCGAATTCCCGGGGATCCATGGCGACGACCTTAAGCAG
pGBD -*14-3-3 κ* R	CTGCAGGTCGACGGATCCTCAATATGCGAGTTTCTGAT
PSP-YCE-*EIN3* F	CGCGCCACTAGTGGATCCATGATGTTTAATGAGATGGG
PSP-YCE-*EIN3* R	AGTACTATCGATGGATCCGAACCATATGGATACATCTT
PSP-YCE-*EIL1* F	CGCGCCACTAGTGGATCCATGATGATGTTTAACGAGAT
PSP-YCE-*EIL1* R	AGTACTATCGATGGATCCGAACCATATTGATACATCTT
PSP-YNE-*14-3-3 λ* F	CGCGCCACTAGTGGATCCATGGCGGCGACATTAGGCAG
PSP-YNE-*14-3-3 λ* R	AGTACTATCGATGGATCCCATAGAGTAGTAATAACTCA

PSP-YNE-*14-3-3 κ* F	CGCGCCACTAGTGGATCCATGGCGACGACCTTAAGCAG
PSP-YNE-*14-3-3 κ* R	AGTACTATCGATGGATCCATATGCGAGTTTCTGATGAT
EIN3-GFP-pCAMBIA1300S F	CTCGAGAAGCTTGGATCCATGATGTTTAATGAGATGGG
EIN3-GFP-pCAMBIA1300S R	CTCTAGAGGATCGGATCCTTAGAACCATATGGATACAT
EIL1-GFP-pCAMBIA1300S F	CTCGAGAAGCTTGGATCCATGATGATGTTTAACGAGAT
EIL1-GFP-pCAMBIA1300S R	CTCTAGAGGATCGGATCCTCAGAACCATATTGATACAT
14-3-3 λ-FLAG-pCAMBIA1300S F	aaatctatctctctcgagATGGCGGCGACATTAGGCAG
14-3-3 λ-FLAG-pCAMBIA1300S R	GTCCTTATAATCctcgagCATAGAGTAGTAATAACTCA
14-3-3 λ-promoter-pCAMBIA1300 F	ctcggtacccggggatccATATAGTCAACACTGAAGCC
14-3-3 λ-promoter-pCAMBIA1300 R	gtcgactctagaggatccTTACATAGAGTAGTAATAAC
EIN3-FLAG-pCAMBIA1300S F	ctcggtacccggggatccATGATGTTTAATGAGATGGG
EIN3-FLAG-pCAMBIA1300S R	ctctagaggatcggatccGAACCATATGGATACATCTT
pGreenII-62SK-*14-3-3 λ* F	TGGCGGCCGCTCTAGAATGGCGGCGACATTAGGCAG
pGreenII-62SK-*14-3-3 λ* R	GCAGCCCGGGGGATCCTCAGGCCTCGTCCATCTGCA
pGreenII-62SK-*EIN3* F	TGGCGGCCGCTCTAGAATGATGTTTAATGAGATGGGAATGTG
pGreenII-62SK-*EIN3* R	GCAGCCCGGGGGATCCGAACCATATGGATACATCTTGCTGC
pGreenII-62SK-*EIL1* F	TGGCGGCCGCTCTAGAATGATGATGTTTAACGAGATGGGAATG
pGreenII-62SK-*EIL1* R	GCAGCCCGGGGGATCCTCAGAACCATATTGATACATCTTGCTGC
pGreenII-0800-*ERF1pro* F	CGGGCCCCCCCTCGAGGCCAAAAGAGATACAATGTCCAGG
pGreenII-0800-*ERF1pro* R	ATTAAGCTTGTAGAAAAAATACTCTGTTTCTTGACTAC
1300S-*EIL1*-cLUC F	GGCGGAGGTCAGATCTATGATGATGTTTAACGAGATGGGAATG
1300S-*EIL1*-cLUC R	TGGATCCCCGGGTACCTCAGAACCATATTGATACATCTTGCTGC

1300S-*ERF1*-nLUC F	CGGTACCCGGGGATCCATGGATCCATTTTTAATTCAGTCCCC
1300S-*ERF1*-nLUC R	ACGAGATCTGGTCGACCCAAGTCCCACTATTTTCAGAAG
**ChIP-qPCR**	
ERF1-ChipQ F1	taatagaatttagttgcgtatccg
ERF1-ChipQ R1	caaatgttgaatcgattatccaaa
ERF1-ChipQ F2	tttttgtttgagaaagaagttgaaa
ERF1-ChipQ R2	cattatcctataatcttaggaagc
ERF1-ChipQ F3	atttgcatatcaaaattatctata
ERF1-ChipQ R3	caggattactttggtgtacccaag
ACT7-ChipQ F	CGTTTCGCTTTCCTTAGTGTTAGCT
ACT7-ChipQ R	AGCGAACGGATCTAGAGACTCACCTTG

69
